# Complex adult congenital heart disease on cross-sectional imaging: an introductory overview

**DOI:** 10.1186/s13244-022-01201-y

**Published:** 2022-04-25

**Authors:** Mahdi Saleh, David Gendy, Inga Voges, Eva Nyktari, Monika Arzanauskaite

**Affiliations:** 1 Department of Radiology, Liverpool Heart and Chest Hospital, Liverpool, UK; 2 Department of Congenital Heart Disease and Paediatric Cardiology, University Hospital Schleswig-Holstein, Campus Kiel, Kiel, Germany; 3 Cardiovascular MRI Unit, BIOIATRIKI SA (Biomedicine Group of Companies), Athens, Greece; 4 Cardiovascular Research Center-ICCC, Hospital de la Santa Creu i Sant Pau, IIB-Sant Pau, Barcelona, Spain

**Keywords:** Adult congenital heart disease, Computed tomography (CT), Magnetic resonance imaging (MRI)

## Abstract

Congenital heart disease is the most common group of congenital pathology. Over the past few decades, advances in surgical treatment have resulted in a rising population of adult patients with repaired complex congenital heart disease. Although the quality of life has greatly improved, a significant proportion of morbidities encountered in clinical practice is now seen in adults rather than in children. These patients often have significant haemodynamic pathophysiology necessitating repeat intervention. CT and MRI are excellent imaging modalities, which help elucidate potential complications that may need urgent management. Although imaging should be performed in specialised centres, occasionally patients may present acutely to emergency departments in hospitals with little experience in managing potentially complex patients. The purpose of this article is to provide an introductory overview to the radiologist who may not be familiar with complex congenital heart disease in adult patients. This educational review has three main sections: (1) a brief overview of the post-operative anatomy and surgical management of the most common complex conditions followed by (2) a discussion on CT/MRI protocols and (3) a review of the various complications and their CT/MRI findings.

## Key points

- The population of adult patients with complex congenital heart disease is rising.
- These patients can present to hospitals with little relevant experience.
- CT and MRI are excellent diagnostic modalities in elucidating potential complications.
- There are numerous common complications radiologists should be familiar with.

## Background

Congenital heart disease is the most common group of congenital pathology. Over the past few decades, advances in surgical treatment have improved long-term outcomes in adult patients with repaired complex congenital heart disease. The average age of patients with adult congenital heart disease (ACHD) is rapidly increasing with a 5–6% rate of annual growth. The estimated prevalence in the adult population is 1.4 million in the United States (US) [1]. Although the quality of life in patients with ACHD has greatly improved, a significant proportion of morbidities encountered in clinical practice is now seen in adults rather than in children. These patients often have significant haemodynamic pathophysiology necessitating repeat intervention. Therefore, lifelong follow-up in specialised ACHD centres is mandatory [2]. Although imaging of ACHD patients should be performed in specialised centres, occasionally patients may present in hospitals with little experience in managing potentially complex patients. Computed tomography (CT) and/or magnetic resonance imaging (MRI) are imaging modalities that provide excellent anatomical detail, visualisation of cardiac and extra-cardiac structures, and an accurate diagnosis, which can influence management [3, 4].

The purpose of this article is to provide an introductory overview to radiologists who may not be familiar with complex ACHD. In the first section, the surgical management and post-operative anatomy of the most common complex ACHD conditions (Tetralogy of Fallot (TOF), D-Transposition of the Great Arteries (D-TGA), and single ventricle defects) will be reviewed. For the purposes of this article, complex ACHD refers to these three pathologies. CT and MRI protocols frequently implemented in repaired complex ACHD as well as associated common complications will be discussed. Complications organised by anatomical areas serve to provide a basis for systematically reviewing any CT or MRI study involving repaired complex ACHD. Given the myriad of conditions and complications present within the spectrum of repaired complex ACHD, extra-cardiothoracic complications are beyond the scope of this article.

### Clinical profile of complex ACHD

ACHD may present in two main ways, excluding incidental lesions picked up due to other reasons. Firstly, approximately 10% of ACHD patients may present with unrepaired disease that may have remained undiagnosed or has been previously diagnosed in childhood. The vast majority of these patients have simple ACHD conditions such as septal defects [5].

The second set of ACHD patients who present acutely make up 90% of cases. These are ACHD patients who underwent surgery in childhood and normally have an extensive past surgical history, which is usually documented. The majority of these patients have complex ACHD, and the numbers are increasing as technological and surgical advances afford better survival rates. These patients have significantly altered haemodynamics and a neo-formed cardiovascular circulation due to the complex interventions performed [5]. Their clinical presentation may be due to an indirect consequence of their altered physiology or a disease-specific post-operative complication presenting in adulthood. Common symptoms and clinical findings include chest pain, shortness of breath, arrhythmias and exercise intolerance [5]. As the number of repaired complex ACHD patients continues to rise, it is exceedingly important to obtain as much clinically relevant past surgical history in order to accurately interpret images. In the acute setting, this may not be possible and a good grasp of the post-operative anatomy of repaired complex ACHD and the common complications that may occur becomes essential.

### Complex ACHD

#### Tetralogy of Fallot

TOF is the most common type of cyanotic congenital heart disease with an adult prevalence of 28,000 in the US [6]. The classic constellation of findings includes subpulmonary infundibular stenosis, overriding aorta, ventricular septal defect (VSD), and right ventricular (RV) hypertrophy. The severity of the defects ranges from minimal overriding of the aorta and trivial pulmonary stenosis to almost complete override of the aorta and pulmonary atresia. The anterocephalad deviation of the outlet septum and hypertrophy of the septoparietal trabeculations are important features, which distinguish TOF from pulmonary stenosis and a VSD [7, 8].

**Fig. 1:**
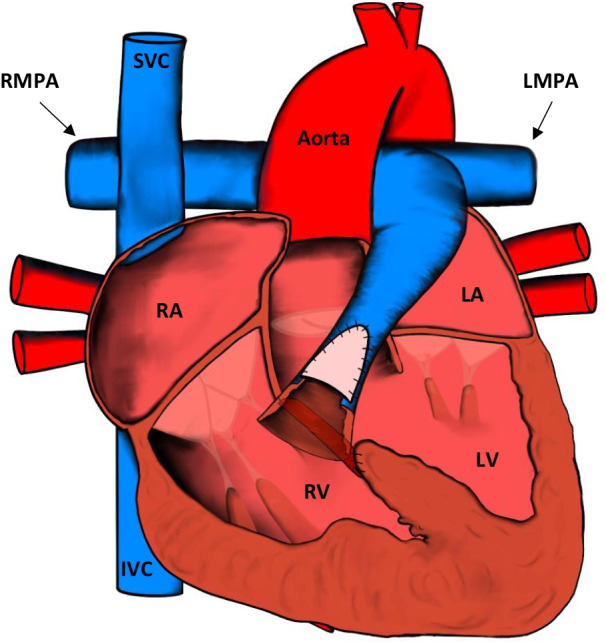
Appearances of the heart after TOF repair. The RVOT is repaired and the VSD is closed. IVC: inferior vena cava; LA: left atrium; LV: left ventricle; LMPA: left main pulmonary artery; RMPA: right main pulmonary artery; RA: right atrium; RV: right ventricle; SVC: superior vena cava

The vast majority of patients will have been diagnosed and undergone surgical correction in the first year of life [9]. The mainstay of surgical treatment for TOF aims to repair the right ventricular outflow tract (RVOT) and close the associated VSD (Fig. 1). Closing the VSD normally involves using a pericardial or synthetic patch, which effectively redirects left ventricular blood flow to the aorta. RV outflow tract repair may entail simple infundibular muscle resection, pulmonary valvotomy, placement of a right ventricle to pulmonary artery conduit, or a transannular patch depending on the severity of the outflow tract obstruction. In patients with severe variations of TOF, associated pulmonary artery stenosis may be corrected by additional patch angioplasty [7, 8, 10].

#### Transposition of the great arteries

TGA has an estimated prevalence of 9000 adults in the US [6]. There are two main categories of TGA: congenitally corrected TGA and D-TGA. Congenitally corrected TGA is a separate and rarer entity, whereby a double discordance exists. There is both an atrioventricular and ventriculoarterial discordance where there is congenital correction of blood flow in the normal direction. Detailed discussion of congenitally corrected TGA is beyond the scope of this article. In D-TGA, where only ventriculoarterial discordance is present, a left-to-right shunt must exist for compatibility with life to occur [11]. D-TGA represents 5–7% of all congenital heart diseases [12].

**Fig. 2:**
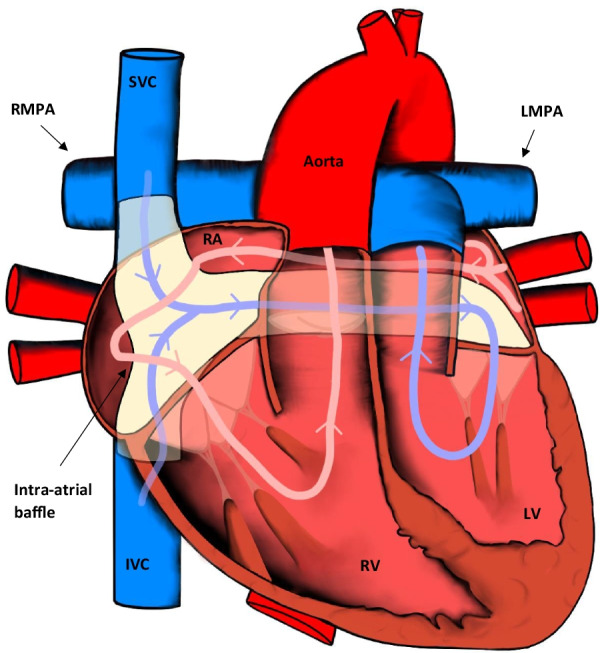
Appearances of the heart after the atrial switch procedure. The SVC and IVC are baffled to the LV, whereas the pulmonary veins are baffled to the RV. IVC: inferior vena cava; LV: left ventricle; LMPA: left main pulmonary artery; RMPA: right main pulmonary artery; RA: right atrium; RV: right ventricle; SVC: superior vena cava

Adults with D-TGA will have had corrective repair during infancy. Initially, atrial switch procedures, or physiologic repair, were the mainstay of treatment. This involved using a two-way baffle to redirect systemic venous return at the atrial level to the left ventricle (LV) and pulmonary venous blood to the RV. Two variants of this procedure exist—the Senning or Mustard technique (intra-atrial channels or baffles produced from autologous and synthetic materials, respectively). The overall aim of the procedure is to create an atrioventricular discordance in order to correct the circulation of the great arteries (Fig. 2) [13, 14].

**Fig. 3:**
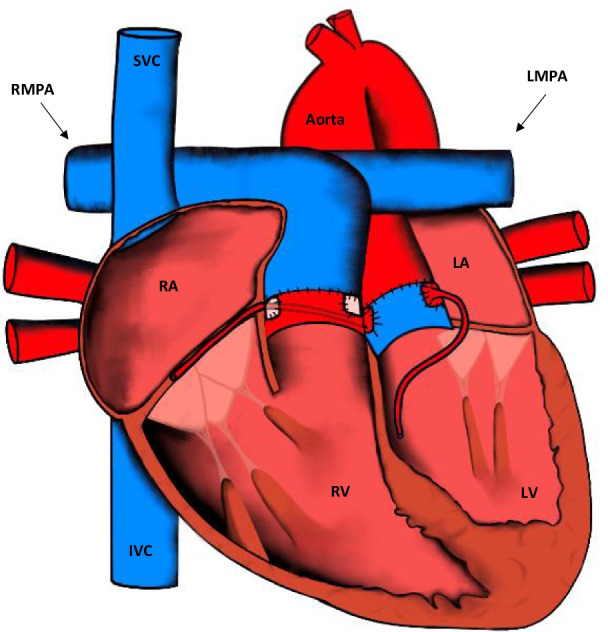
Appearances of the heart after the arterial switch procedure. The aorta and the pulmonary trunk are transected near their origin and translocated along with the coronary arteries. IVC: inferior vena cava; LA: left atrium; LV: left ventricle; LMPA: left main pulmonary artery; RMPA: right main pulmonary artery; RA: right atrium; RV: right ventricle; SVC: superior vena cava

**Fig. 4:**
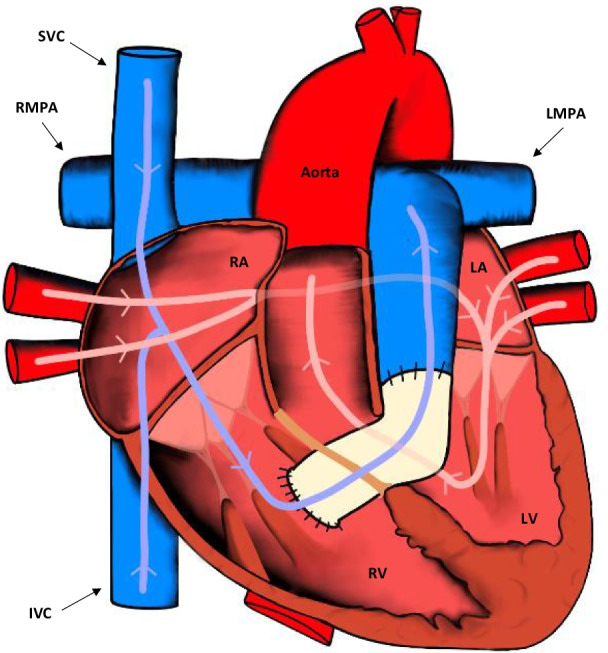
Appearances of the heart after the Rastelli procedure. The VSD is closed and an RV to pulmonary artery conduit is placed. IVC: inferior vena cava; LA: left atrium; LV: left ventricle; LMPA: left main pulmonary artery; RMPA: right main pulmonary artery; RA: right atrium; RV: right ventricle; SVC: superior vena cava

These procedures were superseded by arterial switch procedures, or anatomic repair, in the 1970s as long-term outcomes were better. The aorta and pulmonary trunks are separated. The distal aspects of both arteries are transposed and then anastomosed. Finally, the coronary arteries are translocated to the neo-aorta (Fig. 3). This technique is known as the Jatene procedure [15]. When D-TGA is associated with a VSD and pulmonary valve stenosis, the Rastelli technique is used. This procedure closes the VSD with a patch, which serves as an intra-cardiac baffle, redirecting systemic flow to the aorta. A conduit is then placed between the right ventricular and pulmonary branches to redirect pulmonary flow (Fig. 4) [16]. It is important to recognise that a right ventricle to pulmonary artery conduit can also be placed in patients with pulmonary valve or artery atresia in severe TOF [17].

#### Single ventricle defects

Single ventricle defects can be defined as any anomaly in which there is an abnormal mixture of systemic and pulmonary venous blood associated with atresia or significant hypoplasia of a cardiac chamber. This broad category of congenital heart disease includes hypoplastic left heart, double inlet/outlet ventricular defects, tricuspid atresia, and pulmonary atresia with an intact ventricular septum. Generally, there are several stages of operations performed sequentially during childhood with the objectives of providing continuous systemic outflow, unimpeded venous inflow, and adequate pulmonary blood flow [18].

**Fig. 5:**
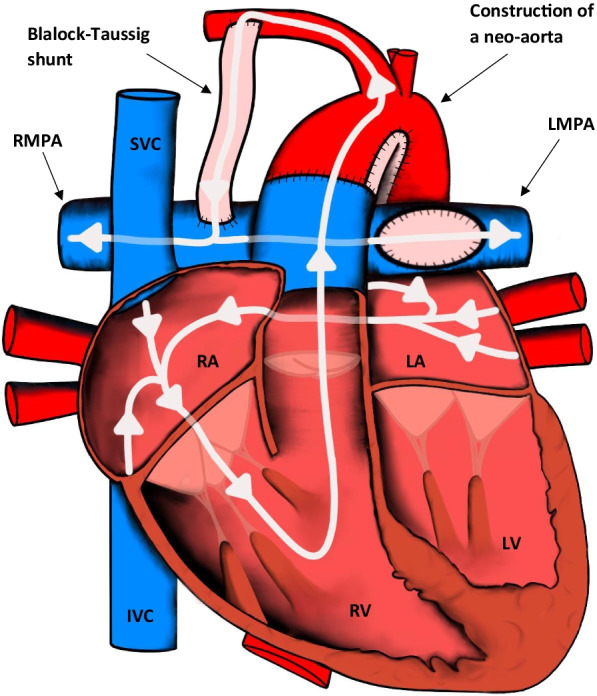
Appearances of the heart after the Norwood procedure (Stage 1), which involves construction of a neo-aorta, atrial septectomy, addition of a modified Blalock–Taussig shunt, and ligation of the ductus arteriosus. IVC: inferior vena cava; LA: left atrium; LV: left ventricle; LMPA: left main pulmonary artery; RMPA: right main pulmonary artery; RA: right atrium; RV: right ventricle; SVC: superior vena cava

In hypoplastic left heart syndrome, which is the prototypical version of a univentricular heart, a three-staged surgical approach that culminates in a Fontan-type circulation has become common practice. The first stage is called the Norwood procedure (Fig. 5), which involves construction of a neo-aorta. This is accomplished by transecting the main pulmonary artery just proximal to its bifurcation and then connecting it to the hypoplastic ascending aorta and aortic arch using a patch repair technique. An atrial septectomy is also performed to allow pulmonary venous blood to reach the RV now responsible for systemic outflow. The patent ductus arteriosus (PDA) is closed, and pulmonary blood flow is maintained via a modified Blalock–Taussig (BT) shunt connecting the subclavian artery with the pulmonary artery ipsilaterally. The aim of this shunt is to provide pulmonary blood flow to obtain a systemic oxygen saturation between 75 and 85%. An alternative to the BT shunt is to create a synthetic conduit between the RV and pulmonary artery instead, which is known as the Sano procedure. These shunts are often removed by the final corrective stage and therefore not seen in adults [18, 19].

**Fig. 6:**
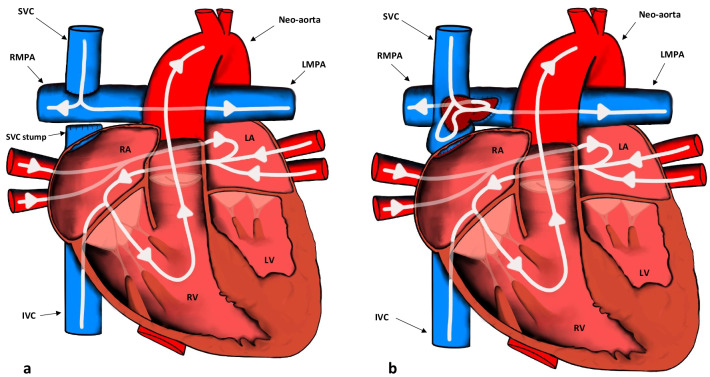
**a** Appearances of the heart after the bi-directional Glenn procedure (Stage 2). Note that the SVC is closed off at or near its junction with the RA in contrast to the hemi-Fontan procedure. IVC: inferior vena cava; LA: left atrium; LV: left ventricle; LMPA: left main pulmonary artery; RMPA: right main pulmonary artery; RA: right atrium; RV: right ventricle; SVC: superior vena cava. **b** Appearances of the heart after the hemi-Fontan procedure (Stage 2). Note that the SVC remains in continuity with the RA in contrast to the bi-directional Glenn procedure. IVC: inferior vena cava; LA: left atrium; LV: left ventricle; LMPA: left main pulmonary artery; RMPA: right main pulmonary artery; RA: right atrium; RV: right ventricle; SVC: superior vena cava

The second surgery (stage 2) aims to create a superior cavopulmonary anastomosis. The goal of this anastomosis is to reduce the preload on the single ventricle by connecting the superior vena cava (SVC) to the pulmonary arteries. The bi-directional Glenn procedure involves dissection of the SVC near or at its junction with the right atrium, closing off the atrial end, and creating an end-to-side anastomosis between the SVC and the right pulmonary artery (Fig. 6a). Of historical note, the no longer used uni-directional or classic Glenn procedure permitted blood flow from the anastomosed SVC to the right main pulmonary artery only, excluding the left main pulmonary artery [20]. In the hemi-Fontan operation (a variation of the bi-directional Glenn procedure), the SVC is connected to the right main pulmonary artery, while its distal aspect remains in continuity with the right atrium. A patch sewn across the superior cavoatrial junction prohibits blood flow into the right atrium from the SVC (Fig. 6b) [21]. The patch is then removed in the last stage of surgery.

**Fig. 7:**
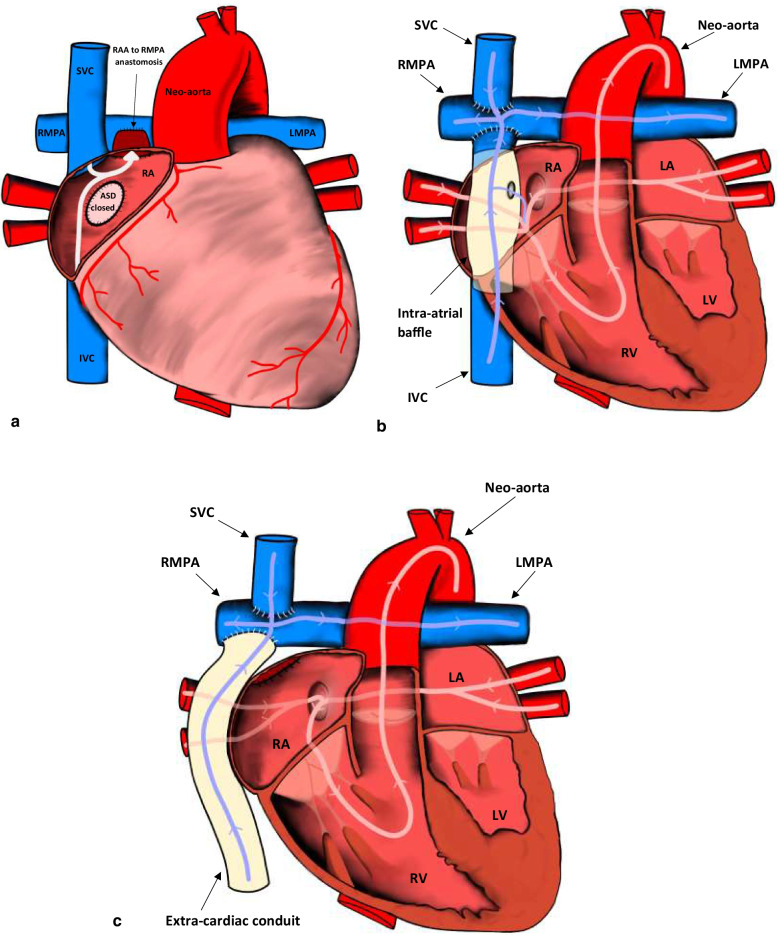
**a** The last and final stage (Stage 3) for definitive repair of single ventricle defects. The IVC is connected to the RMPA to form the atriopulmonary variation of the Fontan procedure. The ASD is closed, and the RAA is anastomosed to the RMPA. ASD: atrial septal defect; IVC: inferior vena cava; LMPA: left main pulmonary artery; RAA: right atrial appendage; RMPA: right main pulmonary artery; RA: right atrium; SVC: superior vena cava. **b** The lateral intracardiac tunnel variation of the total cavopulmonary connection Fontan procedure. A baffle is placed inside the RA, and the SVC is connected directly to the RMPA. A fenestration to relieve pressure in the circuit may be seen between the baffle and the RA. IVC: inferior vena cava; LA: left atrium; LV: left ventricle; LMPA: left main pulmonary artery; RMPA: right main pulmonary artery; RA: right atrium; RV: right ventricle; SVC: superior vena cava. **c** The extracardiac conduit variation of the total cavopulmonary connection Fontan procedure. Note that the conduit that directs venous blood to the RMPA is extracardiac as opposed to the lateral intracardiac tunnel variation. LA: left atrium; LV: left ventricle; LMPA: left main pulmonary artery; RMPA: right main pulmonary artery; RA: right atrium; RV: right ventricle; SVC: superior vena cava

The third surgery (stage 3) is the completion of the Fontan circulation, which allows venous blood returning from the body to flow directly into the lungs, effectively excluding the RV. The main aim is to connect the inferior vena cava (IVC) to the branch pulmonary arteries. These staged procedures may be performed for any of the single ventricle defects. There are three main variations of the Fontan surgery: atriopulmonary (Fig. 7a), lateral intracardiac tunnel (Fig. 7b), and the extracardiac conduit technique (Fig. 7c). Additionally, a fenestration to relieve pressure in the circuit may be seen between the constructed lateral intracardiac tunnel and the right atrium or between the extracardiac conduit and the right atrium [18]. The atriopulmonary variation is not used anymore due to complications described later in the article but can still be seen in older patients.

### CT in complex ACHD

CT is an excellent imaging modality for use in the acute setting due to its rapid acquisition times and high resolution. The latest generation of CT scanners yield a spatial resolution of less than 0.5 mm compared to 0.8–1.5 mm for MRI [22]. A significant cohort of adults with repaired complex congenital heart disease have metallic devices such as coils, stents, and implantable pacemakers, which degrade MRI quality due to susceptibility artefact [23], making CT potentially more favourable. The important factors to consider for CT imaging in complex ACHD can be divided into patient preparation, contrast injection protocols, scan acquisition techniques, and electrocardiogram (ECG) synchronisation. Table 1 provides a summary of CT protocols that can be used for each repaired defect [24].

**Table 1:** Summary of suggested CT protocols by repaired defect

ACHD type	Contrast injection protocol	Range	Sequence	Additional comments
Repaired TOF	Triphasic injection protocol with acquisition timed to aortic opacification	Thoracic inlet to diaphragm	Coronaries or RVOT/pulmonary valve assessment: prospectively ECG gated	Retrospectively ECG gated if irregular rhythm
Repaired D-TGA	Triphasic injection protocol with acquisition timed to aortic opacification A dedicated coronary artery angiogram protocol with ECG gating if assessing the ostia for post-arterial switch procedures	Thoracic inlet to diaphragm	Prospectively ECG gated if assessing the coronary ostia	A triphasic protocol is not suitable for detecting baffle leaks. In this case, a biphasic protocol is advised A delayed phase study is advised if assessing for superior limb baffle obstruction Retrospectively ECG gated if irregular rhythm
Fontan circuit	Options include: Single contrast phase, delayed phase acquisition protocol Biphasic contrast, single delayed phase acquisition protocol Single contrast phase, biphasic acquisition protocol (if coronary imaging needed)	Thoracic inlet to diaphragm Extend to upper abdomen if liver disease is suspected	Prospectively ECG gated if assessment of small structures needed (e.g. for small intracavitary thrombi)	Upper extremity IV access only is most commonly used. Consider upper and lower extremity IV access Retrospectively ECG gated if irregular rhythm

ECG, electrocardiogram; RVOT, right ventricular outflow tract

#### Patient preparation

To image small cardiac structures and to reduce motion artefact, breath holding has become common practice within cardiac imaging [24]. On a similar note, the use of beta blockers to reduce cardiac motion is usual. Ideally, the heart rate should be below 60 beats per minute although guidelines vary by institution. In acute situations, it may be difficult to achieve the desired heart rate. In addition, conditions such as severe pulmonary hypertension and right heart failure limit the use of beta blockers [25]. If detailed coronary imaging is required, sublingual nitroglycerin should be used to increase coronary lumen diameters to improve diagnostic accuracy. However, its use is limited in patients using a phosphodiesterase type 5 inhibitor [24, 26]. If there are no contraindications, both a beta blocker protocol and nitroglycerin should be used to obtain high-quality images, especially if the coronary arteries need interrogating in the setting of potential coronary ostial stenosis post-arterial switch for D-TGA.

#### Contrast injection protocols

The best location of a peripheral intravenous (IV) line is in the right antecubital fossa. Contrast injection protocols should be adapted according to patient weight. In adults, approximately 1.5 mL/kg injected at a rate of 3–5 mL/s is ideal. To further reduce artefact from undiluted contrast material, a saline bolus chasing protocol should be used. This protocol also increases the injection time, which improves the chances of obtaining adequate contrast enhanced images [24, 27].

A dual- or single-phase injection protocol is suggested for pulmonary or systemic arterial angiography. Dual phase with single acquisition involves contrast administration at a constant rate followed by saline. In patients with repaired TOF or post-arterial switch for D-TGA, simultaneous pulmonary and aortic contrast opacification to visualise right and left heart structures requires a more complex approach such as a triphasic protocol. A triphasic, single acquisition protocol involves administering contrast during the first phase, a 50:50 contrast to saline mix in the second phase, and saline in the third phase all while keeping the injection rate constant. Alternatively, the contrast dosage can be halved so that the first half is administered at the arterial rate, the other half at a slower rate, and then followed by saline [24]. A delayed phase can be added to this protocol if venous evaluation is also required.

**Fig. 8:**
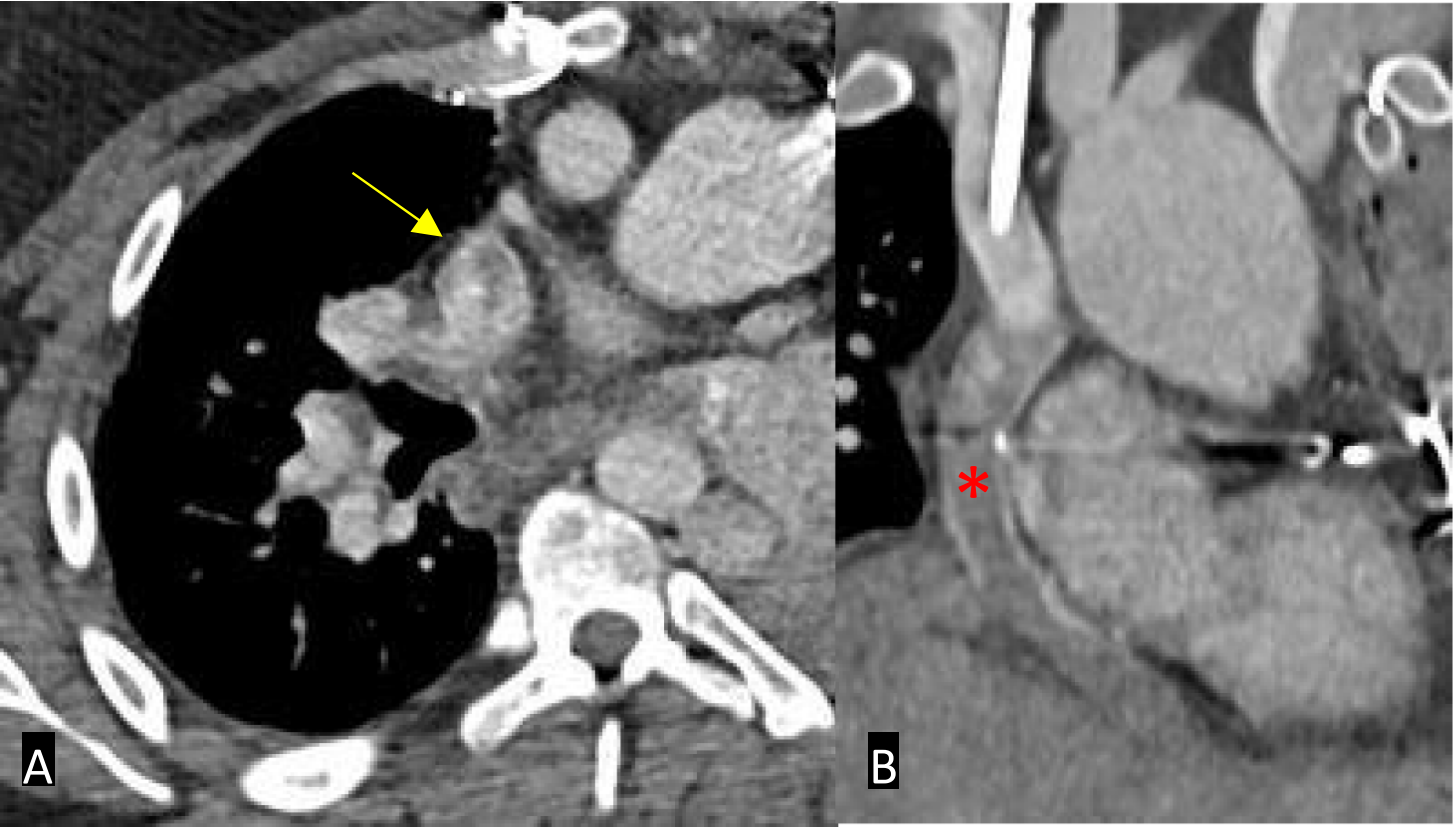
Axial (**A**) and coronal (**B**) contrast-enhanced CT images in a patient with an extra-cardiac conduit Fontan circuit. **A** If the study is protocolled as a standard CT pulmonary angiogram, a pseudo-thromboembolism may be seen – in this case seen in the superior vena cava (yellow arrow). **B** Note that the conduit also appears poorly opacified (red asterisk)

In patients with a Fontan circuit, contrast injection protocols can be more complicated and can vary by institution. If a standard CT pulmonary angiogram protocol is performed in a patient who has a Fontan circuit, a pseudoembolism or pseudothrombus may be demonstrated due to the early acquisition time. The contrast will usually flow into the right main pulmonary artery from the SVC and very little in the left pulmonary artery if the cannula is in the right antecubital fossa. The unopacified blood travels through the IVC or right atrial connection before entering the pulmonary trunk. The poor enhancement of the left main pulmonary artery relative to the well opacified right main pulmonary artery causes a pseudothrombus appearance [28]. Although in our experience, an inappropriately protocolled CT study for thrombus identification may also cause a pseudothrombus anywhere in the circuit due to mixing of opacified and unopacified blood (Fig. 8).

**Fig. 9:**
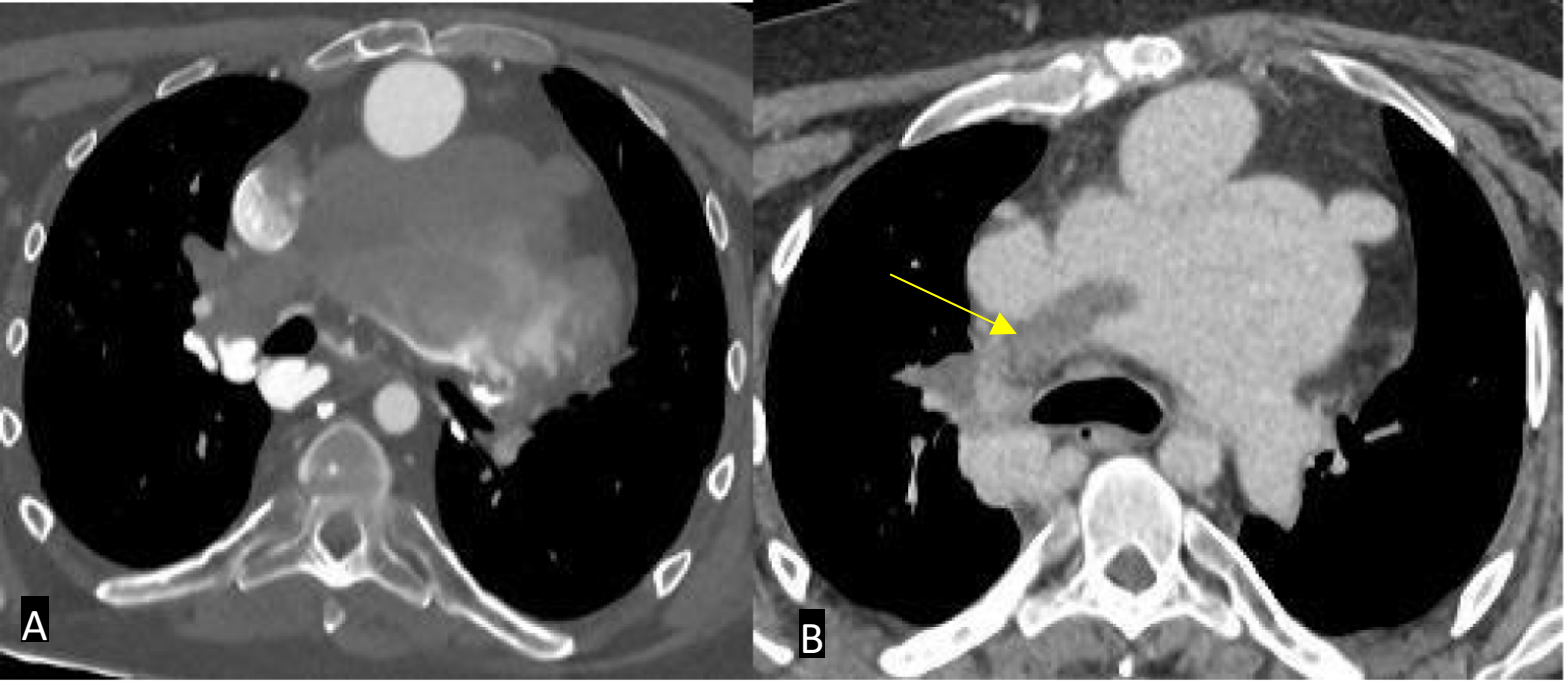
Axial CT images in a patient with an extra-cardiac conduit Fontan circuit. **A** Inadequate protocolling which resulted in suboptimal opacification of the circuit. Note that it is impossible to identify a true thrombus. **B** A single contrast phase, delayed phase acquisition protocol was subsequently used in the same patient which opacified the Fontan circuit adequately and allowed identification of a thrombus in the right main pulmonary artery (yellow arrow)

A cannula in the arm and leg can be considered to rectify this problem although it is not necessary. A single contrast phase, delayed phase acquisition (80–100 s) protocol may sometimes be adequate enough to allow the systemic contrast enough time to return to the IVC and opacify the left main pulmonary artery with the use of more contrast than usual. For example, a total amount of 600–800 mg/kg iodine can be used in this case (Fig. 9). However, a biphasic contrast and single delayed phase acquisition protocol ensures good contrast opacification throughout the Fontan circuit. This advanced protocol also uses more contrast than usually used with a rapid flow rate to ensure good contrast opacification in the delayed phase. Subsequently, the flow rate is decreased to maintain good contrast opacification in the SVC and right main pulmonary artery. A single contrast phase, biphasic acquisition protocol can be used to ensure optimal coronary opacification in addition to the Fontan circuit. The patient is instead scanned twice using a standard dose of iodine (400 mg/kg at 100 kV): once during systemic arterial opacification and once more when contrast has recirculated to allow opacification of the inferior Fontan circuit [24, 27, 28].

#### Scan acquisition timing

Optimal scan acquisition timings can be obtained from bolus tracking or a test bolus. In bolus tracking, a region of interest (ROI) is first determined, which is usually the ascending aorta. After contrast injection, scan acquisition triggers once the predetermined Hounsfield unit (HU) (usually 150) at the ROI has been reached. The bolus tracking technique is suggested only when the cardiac and great vessel anatomy is normal. For lower kV scanning, the HU trigger should be modified and increased accordingly to avoid early scan acquisition caused by the higher contrast opacification [24, 27, 28]. Alternatively, manual bolus tracking can be used in complex abnormal anatomy to trigger acquisition when the operator visually identifies contrast in the area of interest. A test bolus takes into account any pathological systemic pulmonary venous return, intracardiac shunting, or altered haemodynamics such as in Fontan circuits. By injecting a small volume of contrast, around 20 mL, low-dose images can be obtained at several intervals to ascertain the optimal time–enhancement relationship adapted to the patient’s individual contrast haemodynamics [24, 28].

#### ECG synchronisation

ECG synchronisation is not necessary in all patients with repaired complex ACHD. However, ECG synchronisation is advised to reduce motion artefacts or to visualise small intracardiac abnormalities. In retrospective ECG gating, images are obtained continuously throughout the cardiac cycle. The best quality studies are acquired using this method since images can be reconstructed with little motion artefact throughout the cardiac cycle. The major disadvantage is a greater degree of radiation dose exposure compared to prospective ECG gating. Therefore, this method should be limited to patients with significant arrhythmias and patients with elevated heart rates, or when functional data are needed. In prospective ECG triggering, images are obtained at a fixed point or window during the cardiac cycle. This is usually during mid-systole or mid-diastole and is commonly known as the “step-and-shoot” technique. The radiation dose is less using this method compared to retrospective gating. The drawback is that the method is heavily reliant on a regular rhythm [24, 27]. However, methods such as padding, also known as additional surrounding X-ray beam on time, can compensate for a high heart rate and variability [29]. More recent techniques such as dual-source CT imaging allows acquisitions at a high pitch of up to 3.4. This reduces the acquisition time for the chest to less than one second. The result is a significant reduction in movement artefact, which allows for routine non-ECG synchronised studies to be performed in the acute setting. Prospectively ECG triggered high-pitch scanning may also eliminate the need for a breath hold or use of beta blockers [27].

### MRI in complex ACHD

MRI is a good modality for assessing ventricular function and for quantifying blood flow [22]. Specific imaging sequences and protocols frequently implemented in repaired complex ACHD are summarised in Table 2. Generally, an ACHD MRI protocol frequently consists of five main acquisition techniques: anatomic/morphologic imaging, dynamic imaging, blood flow quantification, contrast-enhanced angiography, and tissue characterisation. Images are acquired with ECG-gating, breath-holding, and/or respiratory navigation techniques.

**Table 2:** Summary of MRI techniques and possible protocols in repaired complex ACHD

Technique	Repaired TOF	Repaired D-TGA	Fontan circuit
Bright or black blood imaging	Axial, coronal, sagittal, RVOT, main PAs	Axial, coronal, sagittal, RVOT (if atrial switch)	Axial, coronal, sagittal, main PAs
2D SSFP Cine	LV: 2, 3, and 4 chamber views, short-axis stacks RV: 3 chamber views, RVOT stacks Oblique axial stack of the main PAs	LV: 2, 3, and 4 chamber views, short-axis stacks RV: 3 chamber views, RVOT stacks Oblique axial stack of the PAs (arterial switch) Coronal/axial planes of systemic/pulmonary venous pathways (atrial switch)	LV: 2, 3, and 4 chamber views, short-axis stacks RV: 3 chamber views Short axis and axial stacks covering the whole circuit Coronal stack of the extra-cardiac conduit in long axis
3D SSFP	Detailed assessment of thoracic vasculature	Evaluation of venous baffles (atrial switch) or proximal coronary arteries (arterial switch)	May be more suitable than contrast angiography due to timing of contrast injection issues
Phase contrast	Ao, pulmonary trunk, main PAs	Ao, pulmonary trunk, main PAs IVC/SVC (if atrial switch and obstruction suspected)	Ao, pulmonary trunk, main PAs, cavopulmonary connections, extracardiac conduit
Contrast angiography	Evaluation of main PAs if stenosis suspected	Evaluation of venous baffles (atrial switch) and pulmonary trunk (arterial switch)	Assessment of aortopulmonary collaterals and potential AVMs
Gadolinium enhancement	LV: 2, 3, and 4 chamber views, short axis stacks RV: 3 chamber views, RVOT	LV: 2, 3, and 4 chamber views, short axis stacks RV: 3 chamber views, RVOT	LV: 2, 3, and 4 chamber views, short axis stacks RV: 3 chamber views

Other techniques such as further tissue characterisation by T2-weighted imaging and myocardial mapping can also be considered, if there are relevant indications

Ao, aorta; AVM, arteriovenous malformation; IVC, inferior vena cava; LV, left ventricle; PA, pulmonary artery; RVOT, right ventricular outflow tract; RV, right ventricle; SVC, superior vena cava

#### Anatomic imaging

Anatomic assessment of the heart is fundamentally based on the use of bright- or dark-blood pulse sequences. Dark-blood imaging consists of pulse sequences, which null the blood pool resulting in low signal intensity of fast flowing blood. In turn, this improves visualisation of surrounding cardiac structures. Conventional spin echo sequences, usually used in dark-blood imaging, have been superseded by newer techniques such as turbo spin echo and half-Fourier single-shot turbo spin echo sequences. The advantage of these newer rapid sequences in minimising cardiac motion artefact outweighs the fact that they offer lower signal-to-noise ratio when compared to conventional spin echo sequences. Dark-blood imaging should be performed prior to the administration of gadolinium contrast as gadolinium affects the nulling of the blood signal and results in unwanted artefact. Bright-blood imaging denotes the high signal intensity of fast flowing blood when using associated pulse sequences such as gradient-recalled echo (GRE) sequences or balanced steady-state free precession (SSFP) sequences. Bright-blood imaging is primarily applied to assess cardiac function, but is also frequently used for the assessment of anatomy. The advantages of using SSFP sequences when compared to GRE sequences are that they can be performed rapidly as well as allow higher-contrast and signal-to-noise ratios. Stacks of dark- and bright-blood images are acquired through the target of interest in the axial, sagittal, and coronal planes [22, 30]. Newer bright-blood 3D imaging techniques have emerged allowing excellent depiction of anatomical structures without the use of contrast such as a relaxation-enhanced angiography without contrast and triggering (REACT) sequence, dual gradient echo Dixon, or with a respiratory navigator. These techniques combine the benefits of SSFP imaging with robust background suppression [31].

#### Dynamic imaging

Dynamic imaging consists mainly of cine two-dimensional SSFP imaging. Cine MRI produces a video sequence of the heart in a single predetermined plane. This allows for the evaluation of valvular and ventricular function as well as shunt detection. The standard planes taken vary greatly according to the clinical question, the ACHD type, as well as the type of repair that has taken place. Any combination of short-axis, long-axis, and specific chamber views can be taken according to preference and protocol, which may vary by institution. However, additional planes should be taken to assess for specific circumstances. For example, views through the RVOT or coronal views may be obtained depending on the specific heart defect. Additionally, Cine MRI can be employed to subjectively assess valve movements and to detect regurgitant or stenotic jets at the area of interest [22, 30].

#### Quantification of flow

Blood flow quantification analysis is typically obtained using 2D cine phase-contrast (PC) MRI with velocity-encoded imaging. PC MRI imaging acquires both long- (in-plane imaging) and short-axis planes (through-plane imaging) of vessels and allows flow to be calculated as a product of area and pixel velocity in the through-plane acquisitions. Normally, through-plane imaging is used to calculate flow volumes, including regurgitant fractions and velocities at selected planes, while in-plane imaging is used to detect stenosis along the long axis of the jet in the affected vessel or chamber. Of note, velocity range (encoding) should be appropriately selected for the expected velocity in the vessel: for example, unobstructed venous flow would require encoding of 100 cm/s, while an unobstructed aortic flow would typically have velocity encoding of 150 cm/s. The general recommendation is to use the velocity that is approximately 25% higher than the expected value, and any aliasing artefact should be corrected with encoding adjustments [32]. PC MRI imaging is also the main tool to quantify forward flow and residual intracardiac shunts. PC imaging can assess for differential flow between the main pulmonary arteries in the setting of branch pulmonary artery stenosis. To quantify shunt magnitude, two flow measurements are taken in the through plane: one in the ascending aorta and the other in the pulmonary artery. The pulmonary blood flow (Qp) and systemic blood flow (Qs) are used to calculate pulmonary-to-systemic flow ratio (QP:QS), which represents the direction of shunt and the shunt size if present. A QP:QS greater than 1:1 is interpreted as a shunt from the aortic circulation to the pulmonary circulation, whereas it is vice versa for a QP:QS less than 1:1 [22, 30, 32]. It can also be used in Fontan circulation patients to reliably and objectively quantify systemic-to-pulmonary collaterals (SPC), a method proven to have prognostic value [33]. SPC flow can be calculated by two independent measures: (1) aortic flow minus summed caval flow and (2) summed pulmonary venous flow minus summed pulmonary arterial flow. 4D flow permits visualisation of flow in addition to quantification, and to scan flows at several locations within the field of view simultaneously. This relatively newer technique involves the acquisition of a three-dimensional time-resolved volume sequence with velocity encoding applied in any spatial direction. Additional relevant information such as kinetic energy and wall shear stress can also be assessed [34].

#### Contrast-enhanced angiography and tissue characterisation

Gadolinium contrast-enhanced MRI angiography can clarify aortopulmonary, venovenous collaterals, and arteriovenous malformations. This is especially useful when assessing for small collateral vessels that may be present in certain ACHD conditions such as arteriovenous malformations and SPCs in Fontan circulation patients (discussed in more detail later). The dose of contrast varies; however, 0.1 mmol/kg is adequate for the majority of applications and can be adapted as necessary. Contrast is injected intravenously through a cannula at a variable rate (0.5–4.0 mL/s). Conventional three-dimensional imaging can be performed with or without ECG triggering. ECG-triggered imaging is preferred to minimise artefact from cardiac motion [22, 30].

For thrombus assessment in ACHD, the best approach is to use an early gadolinium phase (within the first minute after contrast injection) inversion recovery sequence with a long inversion time (500–600 ms) to distinguish between thrombus and tumour. Additional late enhancement imaging, according to circumstance, can be used to assess for fibrosis. Late gadolinium enhancement (10–15 min following administration) of myocardium with an inversion recovery sequence may occur in a subendocardial or transmural pattern, representing infarction or in non-ischaemic patterns, representing fibrosis. The TI is predetermined to ensure that imaging results in myocardium that appears black and areas of fibrosis appear bright [30, 35].

## Anatomical review of complications

### Heart

#### Heart failure

Heart failure is the leading cause of morbidity in ACHD. An isolated rise in natriuretic peptides has been demonstrated in up to 53% of ACHD patients. The hospitalisation rate for symptomatic heart failure is approximately 1.2 per 1000 patient-years [36].

In repaired complex ACHD, RV dysfunction is common. In patients who have had definitive TOF repair, pulmonary valve insufficiency leading to right heart failure is the most common long-term complication. The most significant issue with the atrial switch approach for D-TGA is creating a circuit with the RV and tricuspid valve opposing the systemic circulation. The RV is not morphologically designed to withstand long-standing high pressures; therefore, late RV failure and tricuspid regurgitation are expected in this patient group [37]. In the Fontan circulation for corrected single ventricle defects, flow from the systemic veins to the ventricle is passive due to the excluded RV and lack of two valves in the systemic venous circulation. Low resistance in the Fontan circuit is therefore critical. Any degree of impedance at the level of the pulmonary vessels or valves in addition to rhythm disturbances may lead to significant obstruction to flow. In the long term, this may result in presentation of symptoms of right heart failure [37–39]. It is essential to note that RV failure may accompany LV failure; therefore, a comprehensive assessment must be performed.

Cross-sectional imaging findings of LV heart failure develop in a progressive manner. In stage 1, also known as the redistribution stage, cardiomegaly and an increased artery-to-bronchus ratio may be seen. Stage 2 is characterised by interstitial oedema from leaking fluid into the interlobular interstitium as a result of increased pressure in the capillary bed. Interstitial oedema manifests as peripheral septal (Kerley B) lines and interlobular septal thickening on cross-sectional imaging. In stage 3 alveolar oedema, perihilar groundglass opacity progressing to consolidation with bilateral pleural effusions is characteristic [40]. Isolated right ventricular failure findings include right ventricular dilatation, dilatation of the IVC, and reflux of contrast into the IVC/hepatic veins. However, right and left ventricular failure frequently coexist [41].

**Fig. 10:**
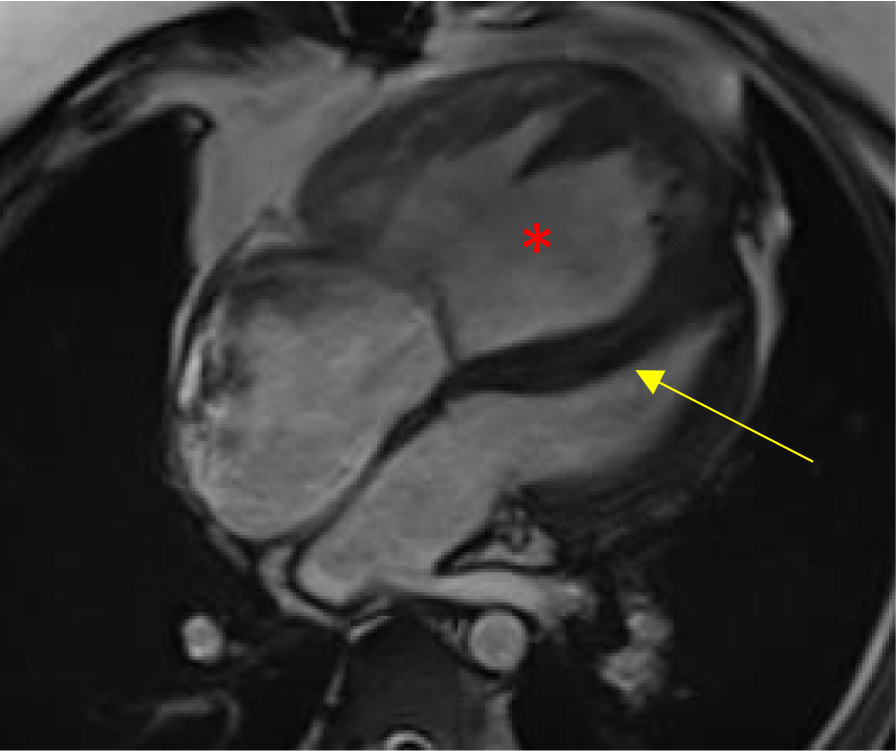
Four-chamber MRI SSFP cine in a patient post-TOF repair. There is significant right ventricular hypertrophy and dilatation (red asterisk). The interventricular septum is also shifted towards the left ventricle (yellow arrow)

**Fig. 11:**
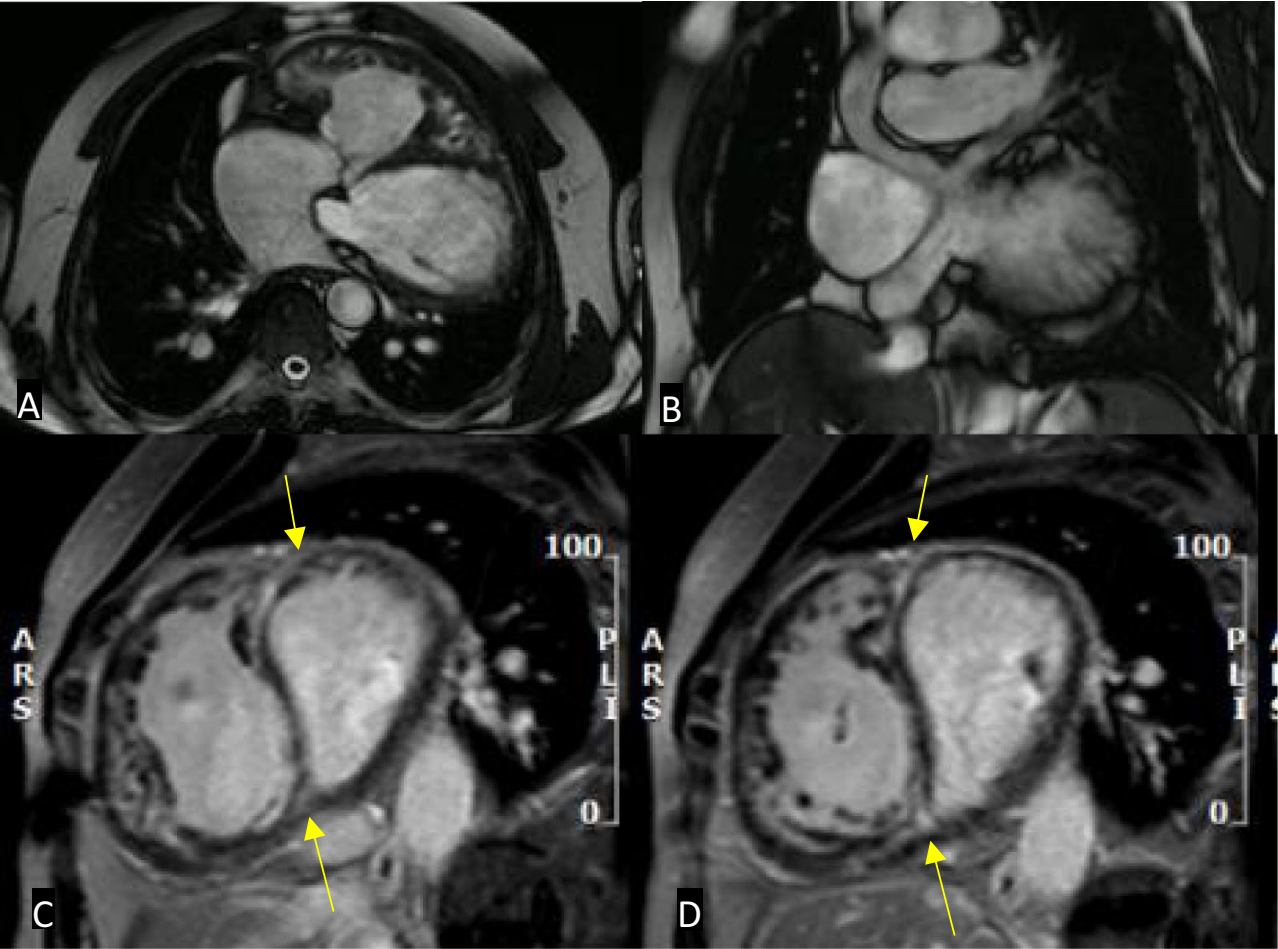
Still shots of MRI SSFP cine in axial (**A**), coronal (**B**) planes and mid-ventricular short-axis views of inversion recovery sequence in the late gadolinium phase (**C**, **D**) demonstrating the appearances of the heart post-atrial switch procedure for D-TGA. **A** Normal appearances of the baffled pulmonary venous system to the right atrium and **B** venae cavae to the left atrium. **C**, **D** Right ventricular dilatation and hypertrophy with late gadolinium enhancement at the insertion points extending into the mid-septum (yellow arrows)

Standard MRI protocols using SSFP sequences provide anatomic and volumetric information in the assessment of suspected congestive cardiac failure including the degree of volume overload. In addition to hypokinetic and dyssynchronous motion of the ventricle(s), other qualitative measures may be scrutinised such as interventricular septal motion. In patients with right heart failure, the interventricular septum may be shifted towards the LV in the context of RV dilatation (Fig. 10). Quantitatively, ventricular volumes and ejection fractions using 2D cine SSFP planes in the ventricular short axis or axial plane should be performed [30]. In repaired complex ACHD, late gadolinium enhancement of the RV is suggestive of fibrosis, which may be seen in chronic RV pressure overload. For example, pulmonary regurgitation or right ventricular outflow tract obstruction in patients with repaired TOF can lead to RV volume overload and late enhancement. Of note, however, surgical history is highly relevant, as RVOT repair patches can appear as regional wall motion abnormalities with late enhancement [42]. Similarly, patients with a Fontan circulation or who have had the atrial switch procedure for D-TGA may demonstrate late enhancement of the RV due to failure (Fig. 11) [43].

### Valves

#### Infective endocarditis

The risk of infective endocarditis (IE) in patients with ACHD is substantially higher than in the general population. In ACHD, the incidence is around 1.1 per 1000 patient-years. IE accounts for up to 4% of admissions to a tertiary ACHD centre, with an associated mortality of approximately 4%. IE may occur in unrepaired, palliated, or corrected defects [44]. Adults with repaired complex ACHD are at the highest risk for developing IE [45].

The pathogenesis in repaired complex ACHD is multifactorial. Mechanical disruption of the valvular or cardiac endothelium due to surgery and the resultant altered flow haemodynamics can trigger the beginning of a significant inflammatory cascade. This response results in the production of inflammatory cytokines, which facilitates bacterial adherence and triggers an immune response. Furthermore, endothelial inflammation activates integrin expression on endothelial cells. Integrins bind circulating fibronectin and promote adhesion of circulating staphylococci. Eventually, vegetation fragments of damaged endothelium can break off and embolise to the lungs and systemic circulation if a residual intracardiac shunt is present [46].

**Fig. 12:**
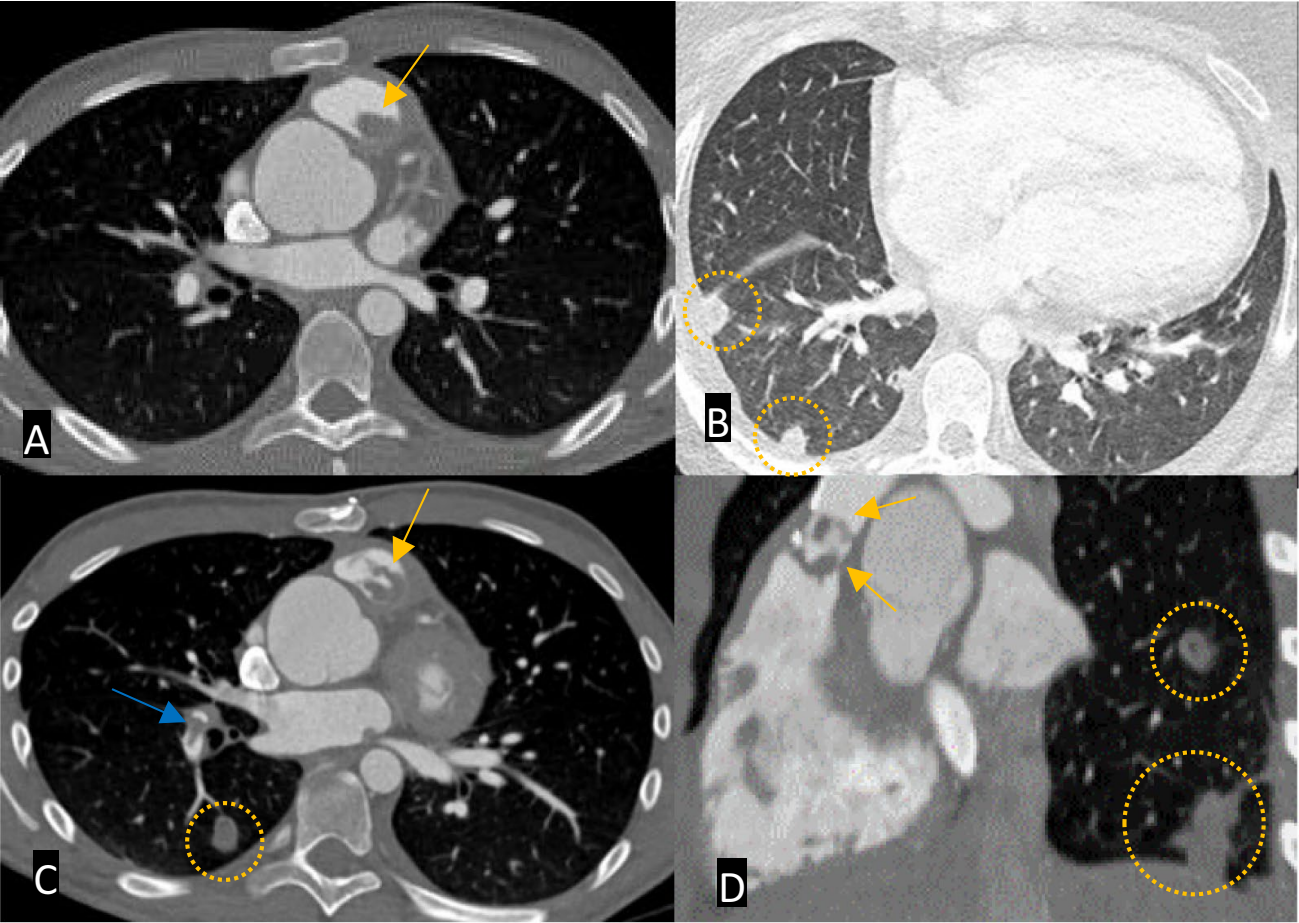
Select multiplanar CT images showing a valved RV-PA conduit to treat RVOT obstruction. Axial images in the bone window (**A** and **C**), lung window (**B**) and MPR reformats in the sagittal plane (**D**), bone window. There are vegetations of the RV-PA conduit (orange arrows), septic emboli in the arteries of the right lower lobe (blue arrow) and pulmonary abscesses (dotted circles). RV: right ventricle; PA: pulmonary artery; MPR: multiplanar reconstruction; RVOT: right ventricular outflow tract

On CT, endocarditic vegetations demonstrate hypoattenuating filling defects surrounded by contrast material. Cardiac CT angiography can also demonstrate the integrity of the valve and any complications such as pseudoaneurysm or fistula formation. If there is progression to septic emboli in the lungs, then the feeding vessel sign, subpleural nodule or wedge-shaped densities with or without cavitation may occur (Fig. 12). The appearance of the often mobile vegetations (usually on a valve or device lead) on MRI ranges from low to intermediate signal intensity on SSFP and inversion-recovery sequences. Post-gadolinium images may demonstrate enhancement of the vegetations and embolic myocardial infarctions. In the absence of vegetations, delayed myocardial enhancement representing endothelial inflammation is also a known finding [47].

### Aorta

#### Aortic dilatation

Significant dilatation of the aortic root is a known phenomenon in complex ACHD even after repair occurs [48]. Although this leads to a higher risk of dissection or rupture, the ten-year incidence of dissection was reported to be as low as 0.2% in repaired complex ACHD [48]. Dissections involving the aortic root should be evaluated using ECG-gated CT angiography, which allows accurate visualisation of the aortic root without significant pulsation artefact. Contrast-enhanced CT may demonstrate a variety of findings such as intimal flap and a double lumen [49].

**Fig. 13:**
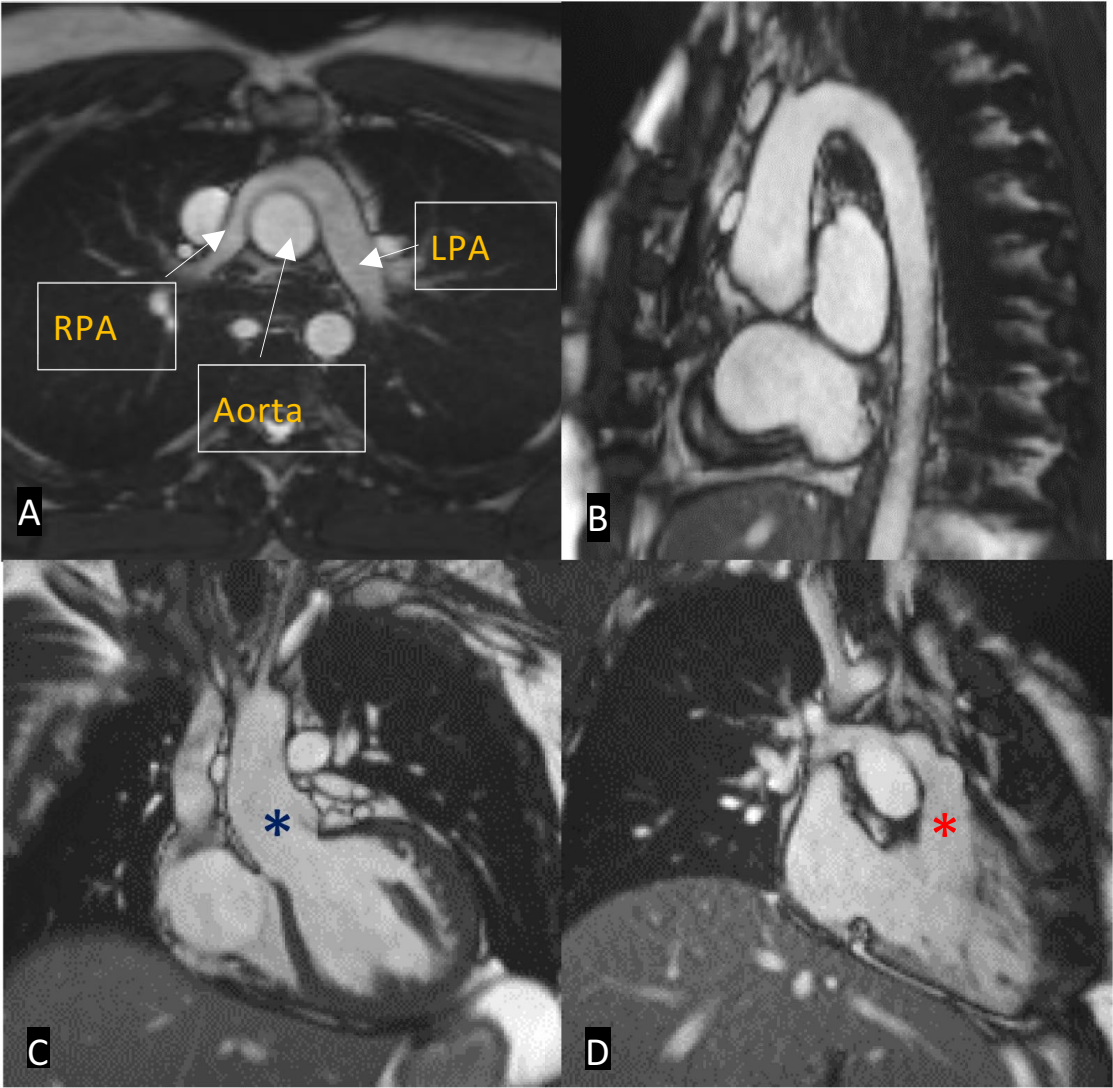
Still shots of MRI SSFP cine in axial (**A**), sagittal oblique (**B**), coronal left ventricular outflow tract (**C**), and right ventricular inflow and outflow (**D**) views post-arterial switch for D-TGA. **A** Typical appearances of the Lecompte manoeuvre for the Jatene procedure. The pulmonary arteries bifurcate anterior to the ascending aorta, classically ‘draping’ over it. **B** Typical appearances of a high aortic arch. **C** Dilatation of the neo-aortic root (blue asterisk). **D** Normal appearances of the neo-pulmonary root (red asterisk). RPA: right pulmonary artery; LPA: left pulmonary artery

The primary intrinsic cause associated with aortopathy in repaired complex ACHD is due to progressive aortic medial degeneration (defined as thinning and fragmentation of the elastic fibres of the aorta), seen in a milder extent than in connective tissue disorders such as Marfan’s. However, specific risk factors in addition to intrinsic medial degeneration are contributory causes. Patients who have had definitive TOF repair are at an increased risk of developing progressive aortic dilatation if there is associated pulmonary atresia and a right-sided aortic arch. Prior pulmonary artery banding and the presence of a VSD are known contributory factors in D-TGA post-arterial switch (Fig. 13). Failure of the Fontan circulation and subsequent development of congestive cardiac failure are strongly associated with significant aortopathy [50].

### Pulmonary vessels

#### Pulmonary hypertension

Pulmonary arterial hypertension (PAH) is defined as a mean pulmonary arterial pressure of 25 mmHg or greater. It is estimated that the prevalence of PAH in ACHD is approximately 10%. Of the five main classifications of PAH, ACHD is classified as Type I (pulmonary arterial hypertension secondary to congenital heart disease) [51].

The pathophysiology of PAH in complex repaired ACHD varies according to the type of condition present as well as the magnitude of any residual systemic to pulmonary shunts that exist. Moderately sized systemic-to-pulmonary residual VSD shunts (post-TOF repair or post-Rastelli repair for D-TGA) can lead to volume overload of the pulmonary circulation and eventual right heart failure as described previously. Muscular hypertrophy, intimal pulmonary artery thickening and the development of plexiform lesions are expected histological findings when PAH has become established [51]. Even after repair without evidence of a residual shunt, patients may still present with PAH several years later. The exact mechanism is still largely unknown, but late diagnosis and closure have been implicated as causative factors [51, 52].

**Fig. 14:**
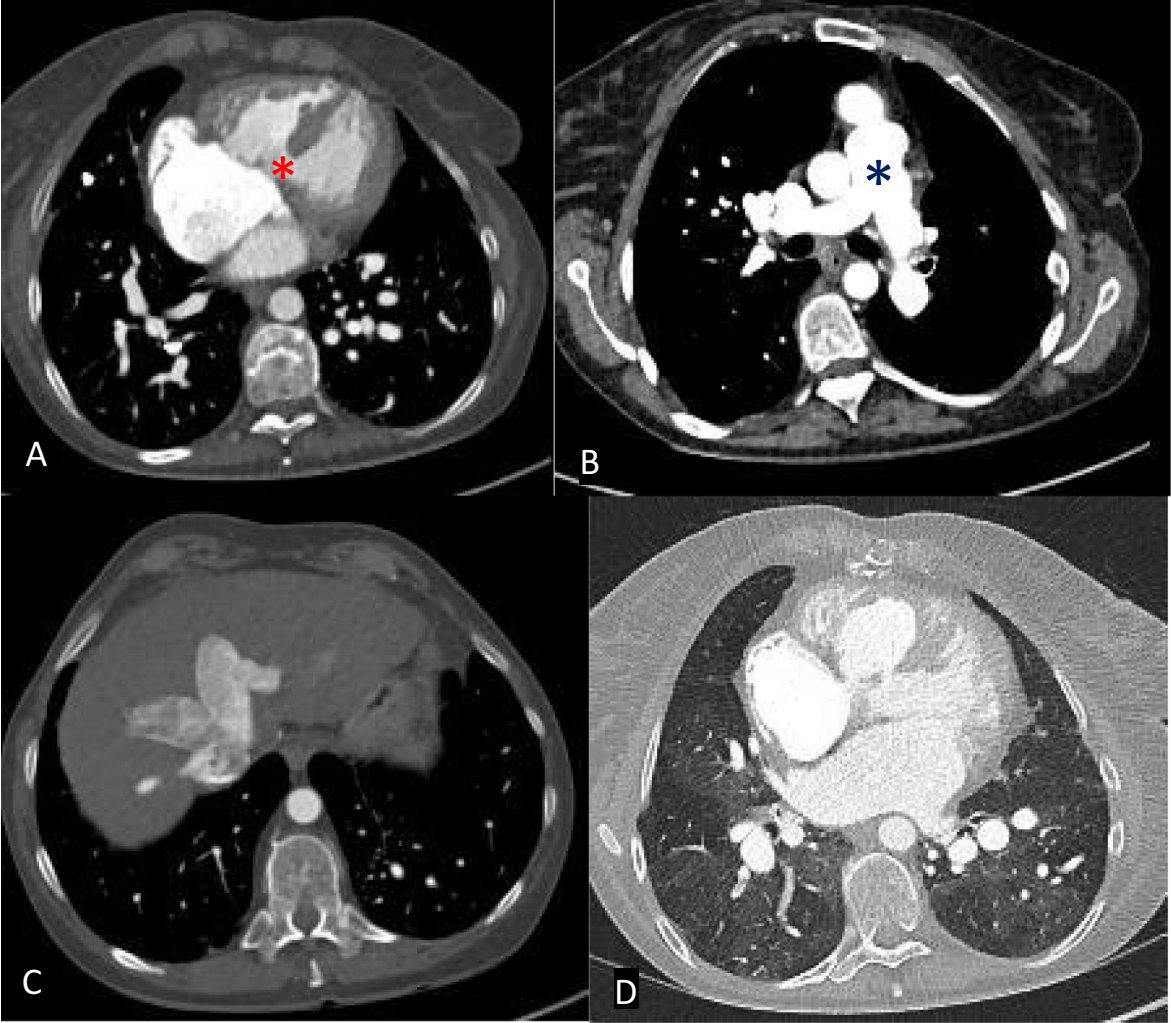
Select axial CT images in the bone (**A** and **C**), soft tissue (**B**), and lung windows (**D**). **A** Large perimembranous VSD (red asterisk) and right ventricular hypertrophy. **B** Dilated pulmonary trunk (blue asterisk) **C** reflux of contrast into the IVC and hepatic veins **D** bilateral pulmonary parenchymal mosaicism. Findings are compatible with pulmonary hypertension and right ventricular heart strain due to a long-standing VSD. Although this is an example of an isolated VSD, similar features of pulmonary hypertension can be seen in large residual VSDs in the setting of repaired TOF or repaired D-TGA with a VSD

Direct cross-sectional vascular findings of long-standing PAH include an enlarged pulmonary trunk, pruning of peripheral pulmonary vessels, and mural calcification. Secondary features that may suggest a degree of PAH includes findings in keeping with right heart strain: RV hypertrophy/dilatation and bowing of the interventricular septum, dilatation and reflux of contrast into the IVC and hepatic veins. Lung parenchymal signs include a mosaic lung attenuation pattern (Fig. 14) [52]. MRI SSFP cine sequences can be used to directly measure the size of the pulmonary trunk and evaluate associated findings such as RV failure. Furthermore, SSFP cine MRI can detect any residual shunts by visualisation of a jet across the area of interest and PC MRI sequences may quantify flow across residual shunts by calculation of the Qp:Qs ratio [30].

#### Collateralisation and arteriovenous malformations

**Fig. 15:**
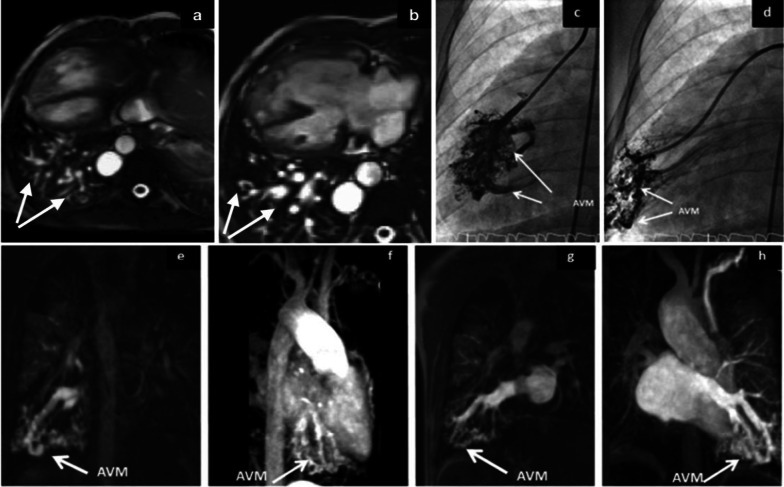
A large right-sided pulmonary AVM in a patient with a Fontan circulation. **a**, **b** Axial still shots of MRI SSFP cine sequences demonstrating a pulmonary AVM in the right lower lobe, **c**, **d** catheter angiogram confirming the presence of an AVM, **e**–**h** contrast-enhanced MRI angiogram in coronal planes demonstrating the AVM (white arrows). AVM: arteriovenous malformation. Re-used with permission from Fontan circulation in an adult: A guide for the radiologist. Arzanauskaite M, Nyktari E, Voges I. ESTI-ESCR 2018 / P-0103

Patients with a significant impendence to flow due to resistance within the Fontan circuit may decompress through arterial or venous collaterals to the lower pressure pulmonary veins or left atrium. Venous collaterals may develop in several locations including the bronchial veins and the venae cavae as well as between the upper-limb veins and pulmonary venous system. Collaterals forming in these areas result in right-to-left shunts, which serve to naturally lower the pressure within the Fontan circuit in order to maintain ventricular preload. These patients are also at increased risk of developing pulmonary arteriovenous malformations. Lack of hepatic venous effluent and the slow non-pulsatile flow state of the Fontan circuit are both considered to be underlying mechanisms in their formation. Pulmonary arteriovenous malformations can occur even before Fontan circuit completion, when the superior cavopulmonary anastomosis is formed due to unilateral hepatic venous flow from caval offset. The primary consequence is decreased systemic oxygen saturation due to right-to-left shunting [18, 31, 37]. Both MRI and CT angiography are excellent in depicting the presence, course, and severity of collateral vessels. CT may demonstrate dilated pulmonary vessels, which extend to the periphery of the lung. Maximum intensity projections (MIPs) using multiplanar reformats aid in visualisation and localisation of collaterals and arteriovenous malformations on CT [53]. Contrast-enhanced MRI angiography acquired as a dynamic sequential sequence may demonstrate vessels better than CT (Fig. 15). Flow quantification from shunts by collaterals and arteriovenous malformations can reliably be assessed with through-plane PC MRI [33, 54].

### Airway and lungs

#### Airway obstruction

Airway obstruction is often the result of the distorted anatomy in ACHD patients who have undergone complex surgical repair or due to the development of complications. Extrinsic compression caused by surrounding vascular structures such as dilated pulmonary arteries or due to cardiomegaly can occur. For example, in patients with repaired TOF, airway stenosis or obstruction may occur secondary to significantly dilated pulmonary arteries proximal to an area of branch pulmonary stenosis. If the left atrium is markedly dilated, splaying of the carina and compression of the main bronchi can be demonstrated [55]. Furthermore, since the left main bronchus is surrounded by important vascular structures such as the pulmonary vessels, chronic airway obstruction from dilated pulmonary arteries can lead to left lung hyperinflation [55]. Nabo et al. detected emphysematous change in 44% of patients with ACHD, of which a subset were patients with VSDs. Segmental emphysematous change was found to be more frequent on the left side than on the right side (14.8% vs. 6.5% of analysed segments) [56]. The pathophysiological mechanism is likely due to significant pulmonary hypertension and dilatation, which can occur from residual VSDs.

#### Plastic bronchitis

**Fig. 16:**
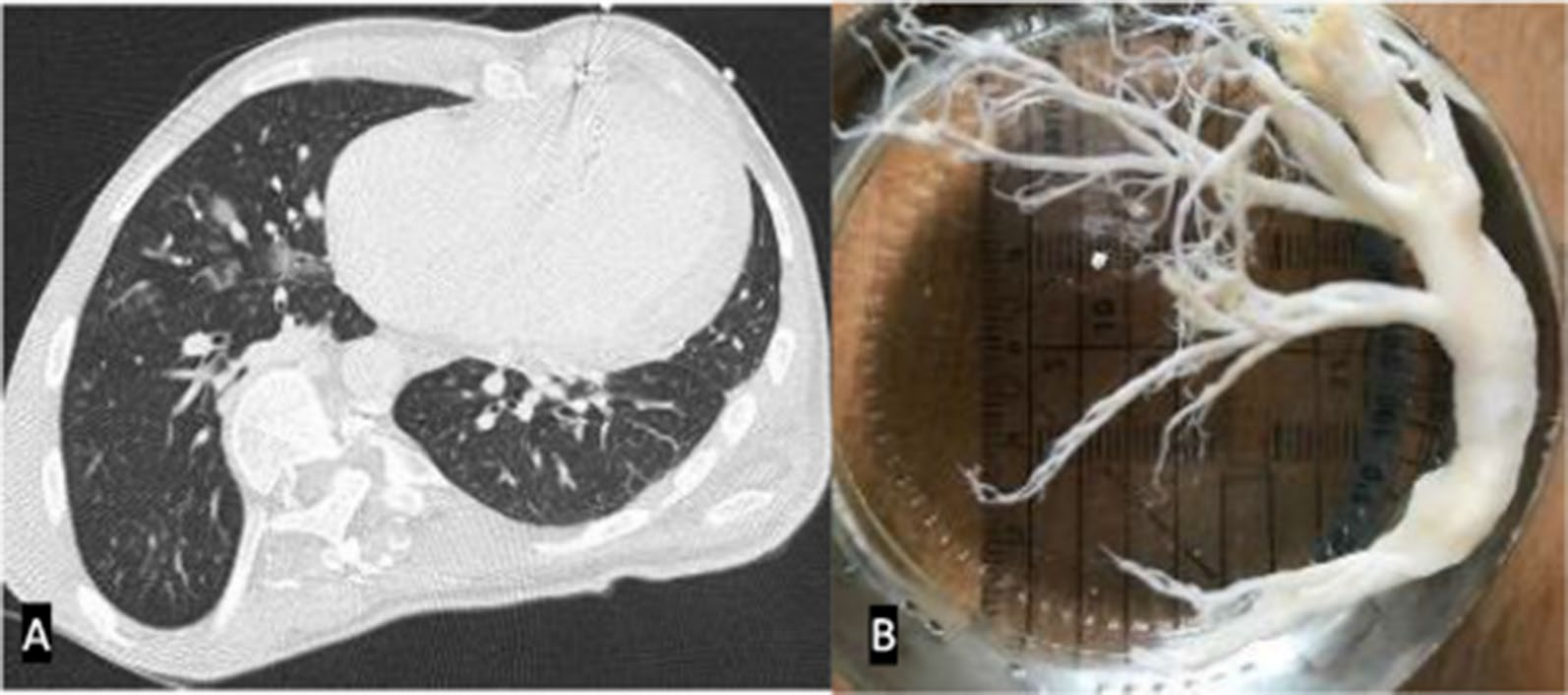
**A** CT Axial image in the lung window in a patient with plastic bronchitis. Note the groundglass parenchymal lung changes in the right upper lobe. **B** A bronchial cast retrieved in a patient with plastic bronchitis

Plastic bronchitis is a relatively rare complication after Fontan correction with less than 1% developing this complication. Onset occurs from as early as one month to two decades after Fontan completion. It is characterised by formation of gelatinous plugs in the large airways that take the shape of bronchial casts due to chronically elevated lymphatic pressures. The acellular casts produce plugs of mucous in the tracheobronchial airway. Intrathoracic pressures increase as a result leading to interstitial pulmonary lymphoedema. Cross-sectional CT imaging often demonstrates indirect signs of plastic bronchitis such as groundglass change, consolidation and/or atelectasis (Fig. 16). Occasionally, bronchial casts in the central airways in the form of filling defects may be seen. Additional findings include a pericardial effusion and/or chylothorax. The affected airways may be completely or only partially compromised by the bronchial casts, resulting in an acute presentation [57].

### Post-surgical complications and neo-pathways

#### Repaired TOF

**Fig. 17:**
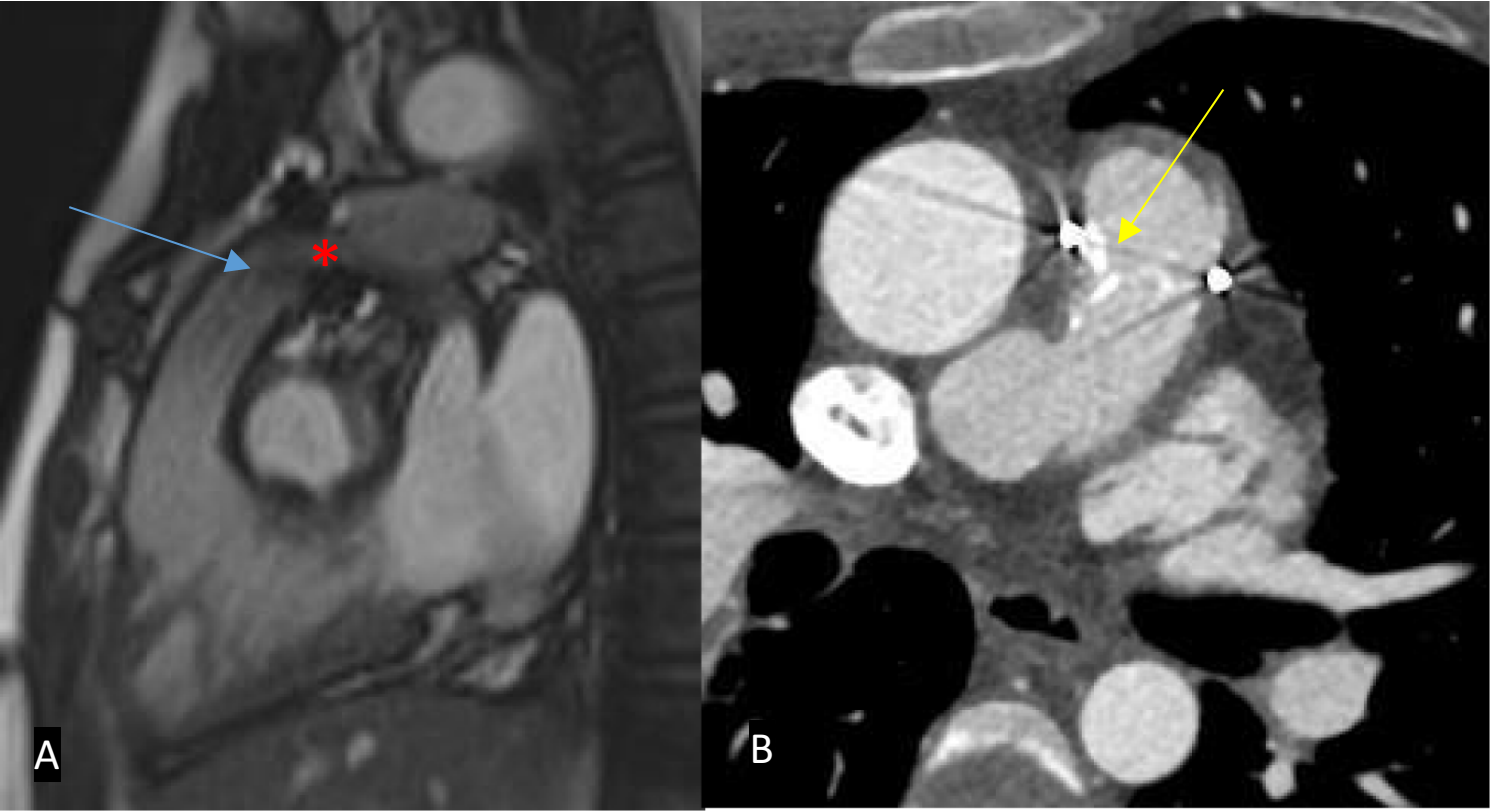
MRI SSFP cine (**A**) and contrast-enhanced CT (**B**) images in a patient with a valved (red asterisk) RV-PA conduit. **A** Single shot of the RVOT demonstrating pulmonary regurgitation (blue arrow). **B** Single axial image demonstrating calcification around the valve (yellow arrow). RV: right ventricle; PA: pulmonary artery; RVOT: right ventricular outflow tract

There are several specific complications which may be seen in adults who have undergone surgical correction for TOF as children, including residual RVOT obstruction, pulmonary regurgitation, residual pulmonary artery stenosis, residual shunts (commonly VSD), and re-entrant atrial arrhythmia potentially resulting in thrombosis. A complication associated with transannular patch repair in TOF is aneurysmal dilatation of the RVOT. In patients who have had a RV to pulmonary artery conduit placed, additional complications such as conduit stenosis, calcification, and regurgitation should be actively considered before issuing a report (Fig. 17) [42].

**Fig. 18:**
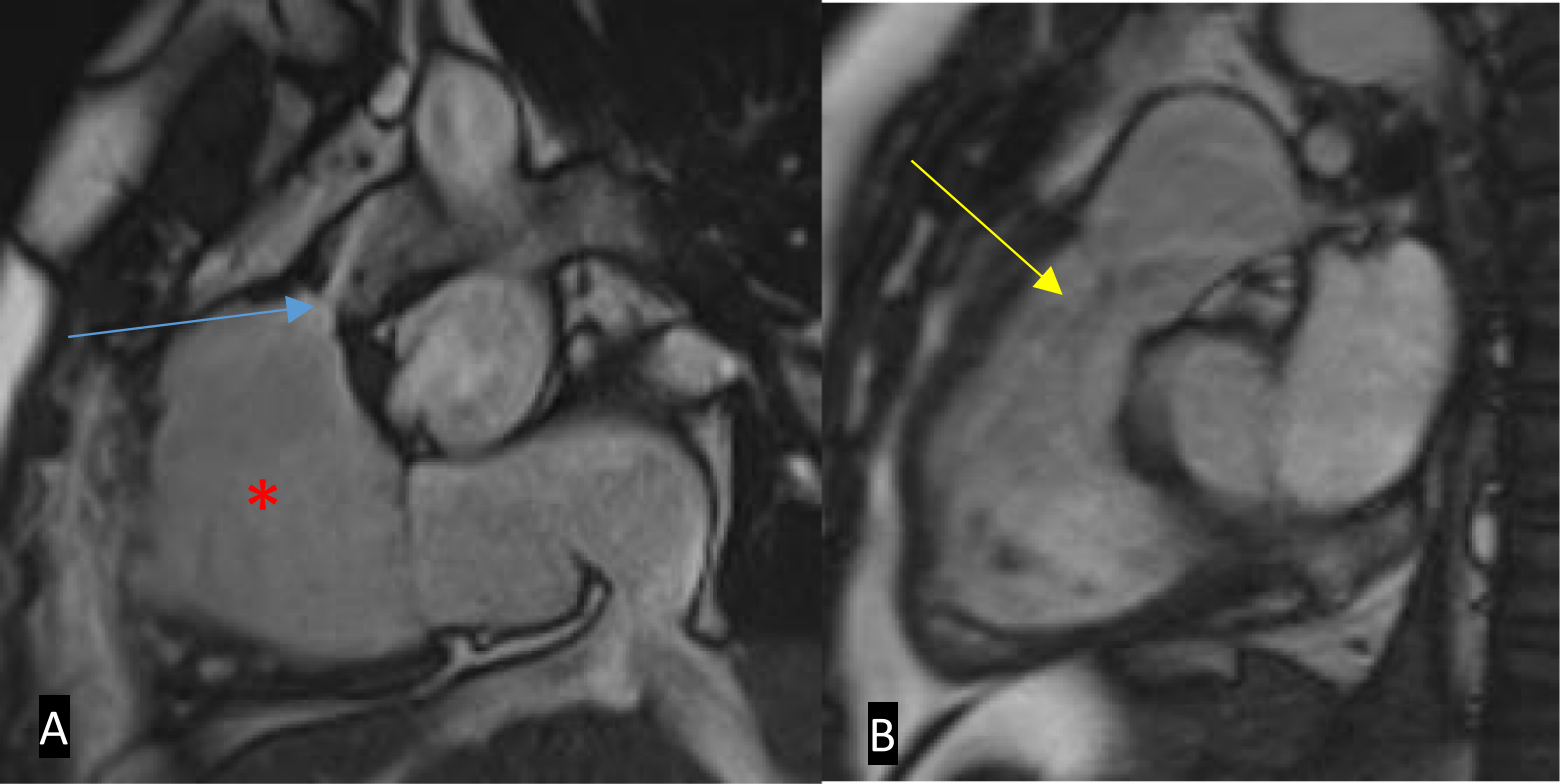
MRI SSFP cine images in two different patients post-TOF repair taken in the RVOT plane. **A** There is RVOT residual obstruction with a stenotic jet (blue arrow) with RV dilatation (red asterisk). **B** A jet of pulmonary regurgitation can be seen (yellow arrow). RVOT: right ventricular outflow tract; RV: right ventricle

**Fig. 19:**
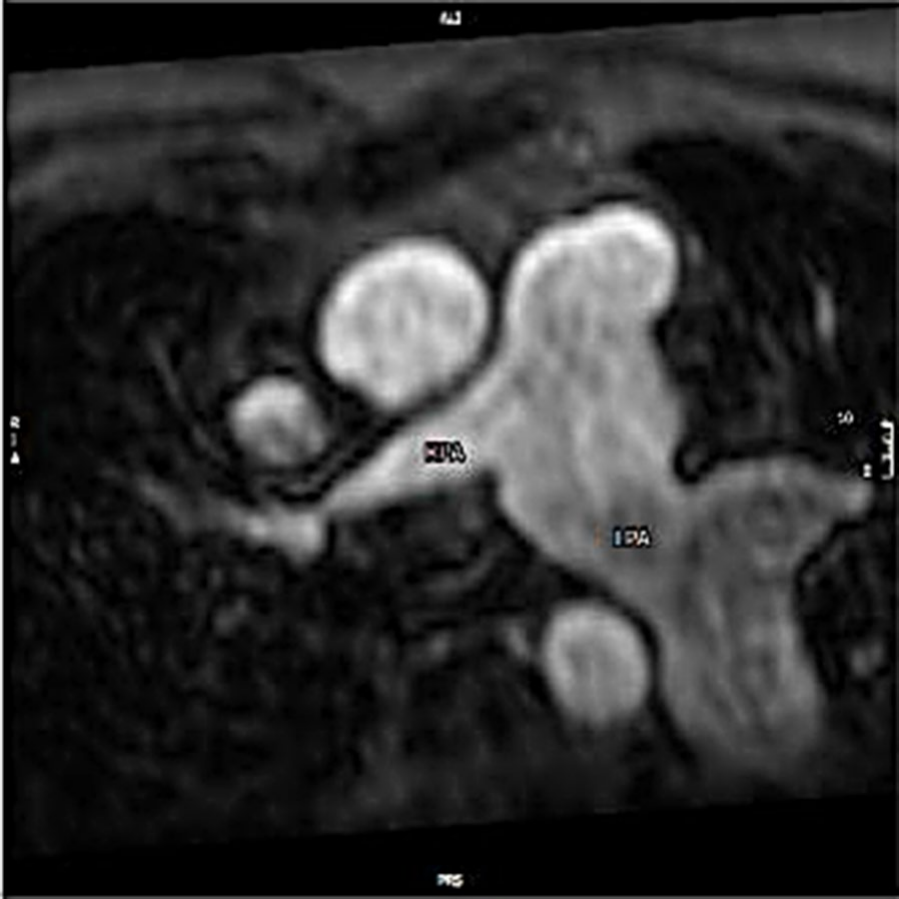
Still shot post-contrast MR angiogram in the axial view. Residual right branch pulmonary stenosis after TOF repair. RPA: right pulmonary artery; LPA: left pulmonary artery (dilated)

2D SSFP cine MRI, which is in plane with the RVOT, in addition to the standard planes, should be acquired to assess for RVOT obstruction, aneurysmal dilatation, or a pulmonary regurgitant jet and through the conduit (if present) to assess for stenosis (Fig. 18). Additional post-contrast MR angiography imaging of the pulmonary arteries allows direct visualisation of potential branch pulmonary artery stenosis (Fig. 19). Proximal stenosis of either of the main pulmonary arteries or conduit can be further assessed on MRI angiographic images or 3D SSFP sequences [31, 37, 38]. Quantification of pulmonary regurgitation and branch pulmonary artery/conduit stenosis can be assessed with through-plane and in-plane PC sequences, respectively. If necessary, additional through-plane PC images through the main pulmonary arteries can demonstrate differential flow into the lungs. The Qp:Qs ratio should be calculated if the suspicion for a residual shunt is high. Delayed enhancement suggestive of fibrosis in addition to the expected enhancement of the VSD or transannular patch is a sign of long-standing dysfunction and may have a prognostic value [42].

#### Repaired D-TGA

**Fig. 20:**
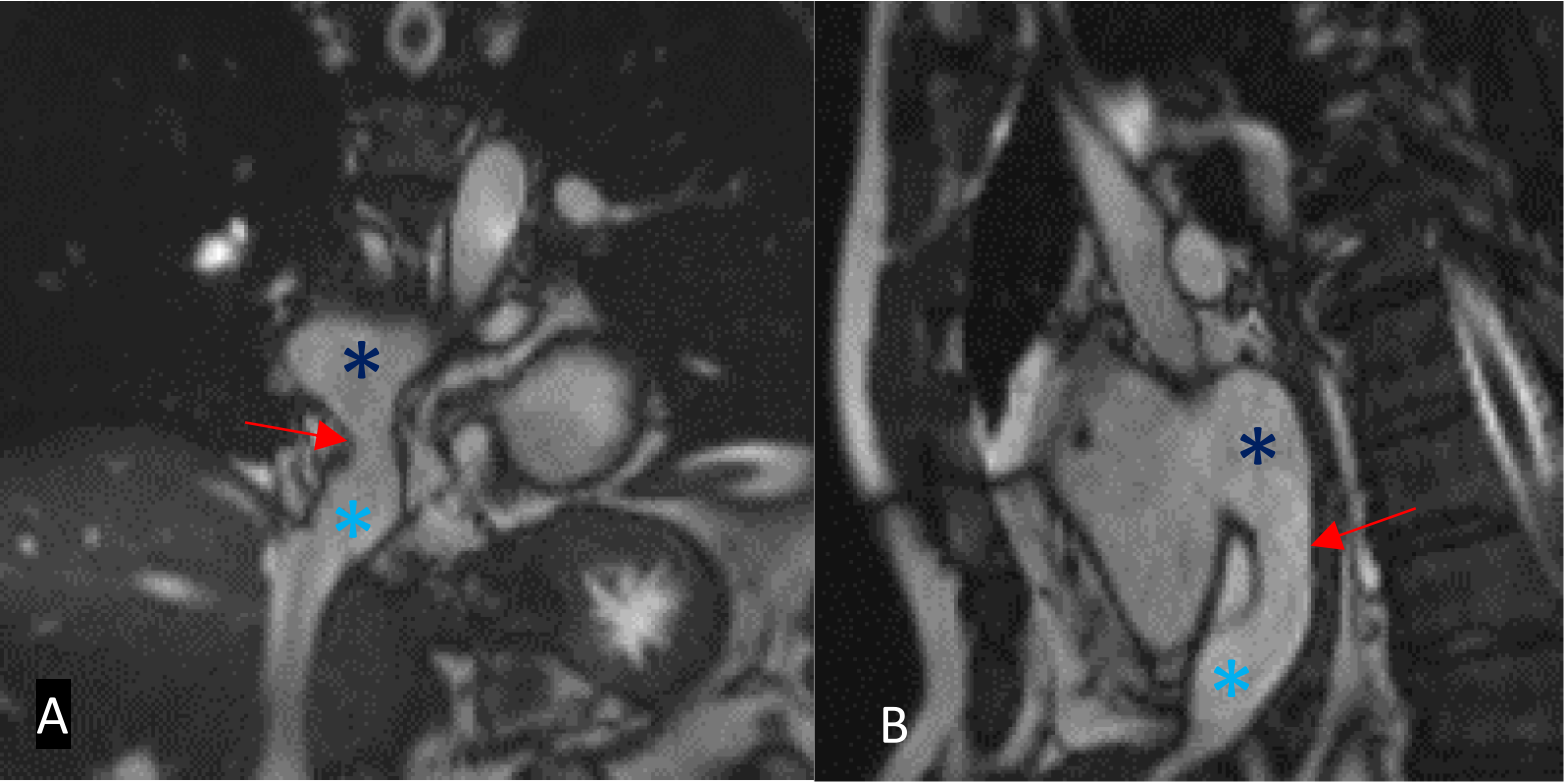
Still-shot MRI SSFP cine images in the coronal (**A**) and sagittal (**B**) plane post-atrial switch for D-TGA. Baffle leak representing a large and broad tunnel from the IVC to the right atrium with a right-to-left shunt (Qp/Qs = 0.7). Dark blue asterisk: pulmonary venous channel; light blue asterisk: IVC channel; red arrow: baffle leak

The intra-atrial baffles created during the atrial switch procedure for D-TGA may become significantly narrowed or occluded as the patient grows. Baffle stenosis is more commonly seen in the superior vena cava limb, which is thought to be due to the constricted anatomy between the baffle and right atrial appendage. Small leaks in the constructed baffles, which most commonly present as a left to right abnormal flow, may also be present. Often, these are haemodynamically insignificant and can be compared to small shunts that occur in septal defects. CT can help detect stenotic areas and visualise leaks; however, it cannot quantify flow or severity. Stacks of coronal oblique and short-axis SSFP Cine or angiographic MRI images through the atria can allow for detection of baffle leaks and stenosis (Fig. 20). Further quantification of peak velocity through stenotic areas can be obtained with in-plane PC MRI imaging [58]. Additionally, small baffle leaks may be difficult to directly visualise; therefore, the calculation of the Qp:Qs is useful [59].

**Fig. 21:**
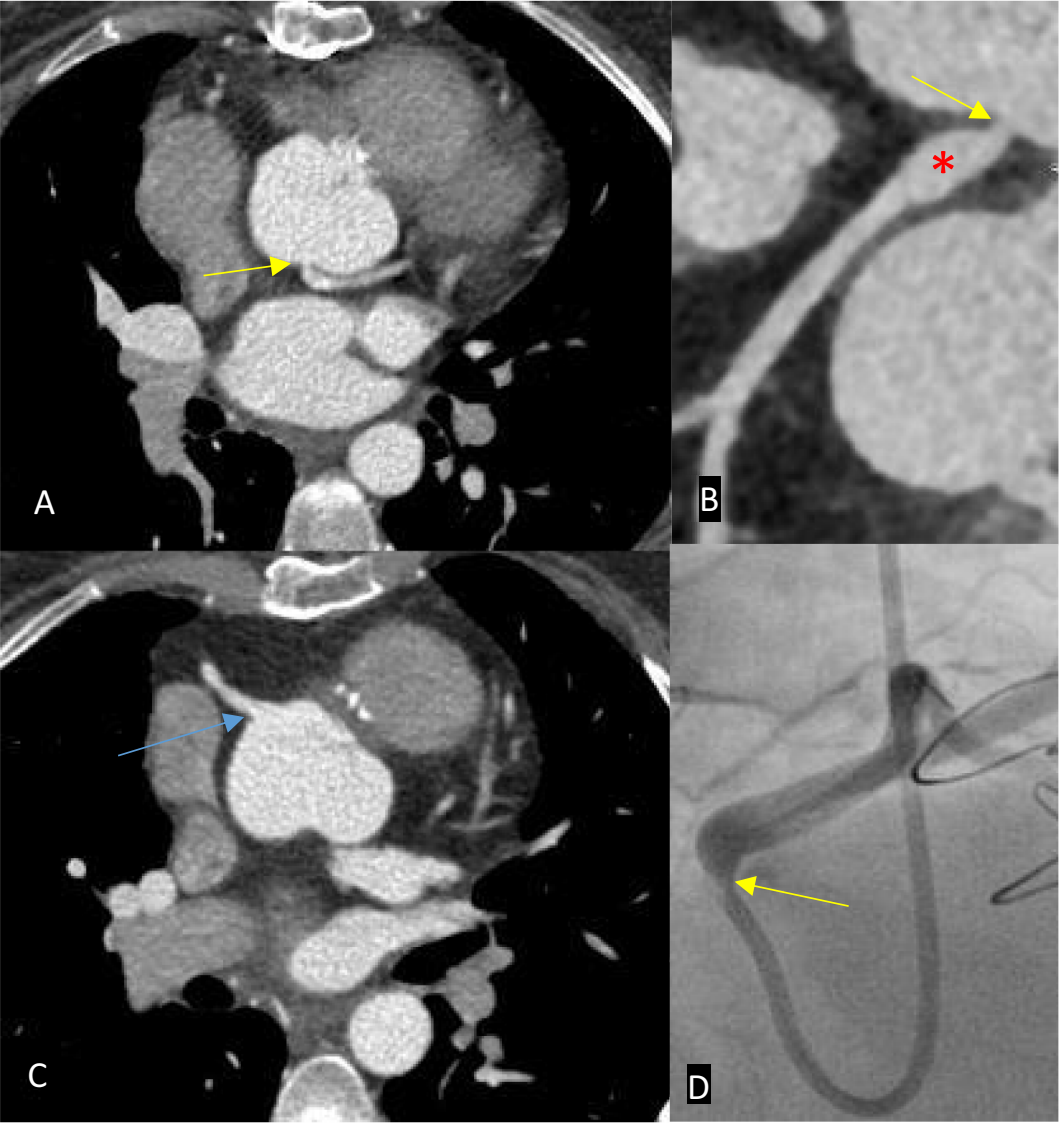
Select coronary CT angiography images (**A**, **B**, **C**) in a patient post-coronary artery translocation. **A** Axial view showing ostial stenosis of the LCA (yellow arrow). **B** Reconstructed view of the LCA showing ostial stenosis (yellow arrow) and post-stenotic dilatation (red asterisk). **C** Axial view showing the normal ostium of the RCA (blue arrow) for comparison. **D** Catheter angiogram of the LCA showing ostial stenosis (yellow arrow). LCA: left coronary artery; RCA: right coronary artery

In patients who have had an arterial switch procedure, the most common complications are supravalvular and branch pulmonary artery stenosis (left more common than right) due to post-surgical manipulation of the pulmonary trunk to anastomose it to the former aortic root. There is also a considerable risk of coronary ostial stenosis and resultant ischaemia due to coronary artery translocation during surgery. Neo-aortic root dilatation may result in significant aortic regurgitation. Assessment of ventricular function using standard 2D SSFP cine sequences through the heart and both ventricular outflow tracts in addition to stack images in the short axis allows for adequate visualisation of potential complications and regurgitant/stenotic jets. Specifically, SSFP cine MRI oblique axial stacks through the main pulmonary arteries can be useful in detecting stenotic areas, and 3D SSFP sequences or a dedicated CT coronary angiogram can assess for coronary ostial stenosis (Fig. 21) [38, 58]. Flow quantification using PC MRI in the through plane to assess for aortic regurgitation in the setting of neo-aortic root dilatation should be performed as well as in-plane imaging at any stenotic areas to assess for peak velocity [31, 37, 38]. In cases of post-Rastelli procedure for D-TGA with VSD and pulmonary stenosis, the main complications are related to the RV to pulmonary artery conduit as previously discussed.

#### Fontan circuit

**Fig. 22:**
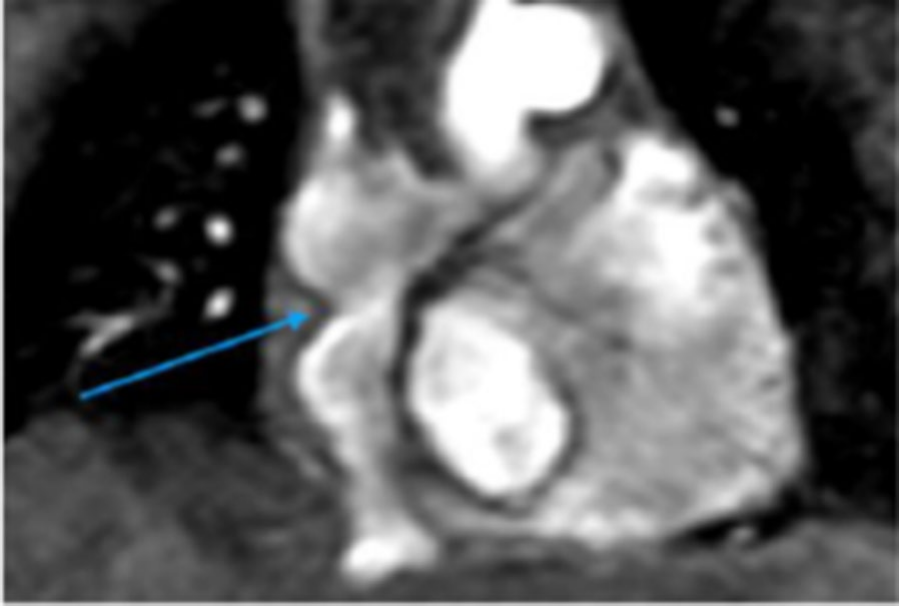
Still-shot MRI SSFP cine in the coronal view. Stenosis within the intra-atrial tunnel in a patient with a Fontan circuit (blue arrow). Re-used with permission from Fontan circulation in an adult: A guide for the radiologist. Arzanauskaite M, Nyktari E, Voges I. ESTI-ESCR 2018 / P-0103

Specific complications related to the Fontan circuit, whether it involves the extra-cardiac conduit or a lateral tunnel baffle used in the modified Fontan procedure, are due to the breakdown and growth-related senescence of these pathways. Thrombosis, leak, and stenosis are known complications (Fig. 22) [37]. The presence of an atrial thrombus is more commonly seen in older patients with the atriopulmonary variation of the Fontan procedure and can be detected with either CT or MRI. Dilatation of the right atrium and slow flow within it can occur and may result in venous obstruction, which facilitates thrombus formation [37–39]. 2D cine and 2D PC MRI for evaluation of the single ventricle and valvular function as well as flow through the cavopulmonary anastomoses can be performed, respectively. In this context, 2D cine SSFP images in different orientations are performed to assess ventricular function and to evaluate for the patency of the Fontan pathway. Quantification of flow using PC MRI is very useful in elucidating areas of stenosis or obstruction that may not be immediately apparent on the SSFP sequences. Furthermore, the PC sequences can be employed to quantify any variations in pulmonary blood flow to the right and left pulmonary arteries. For a complete quantitative flow assessment, points of interest can be placed in the aorta, pulmonary arteries, venae cavae, and pulmonary veins. The choice of planes to use greatly varies among operators and by institution, depending on the individual case scenario in terms of anatomy and haemodynamics. Well-oriented images including the use of sagittal oblique planes to help visualise the necessary parts of the Fontan circuit should be acquired to help answer the clinical question [31, 38].

**Fig. 23:**
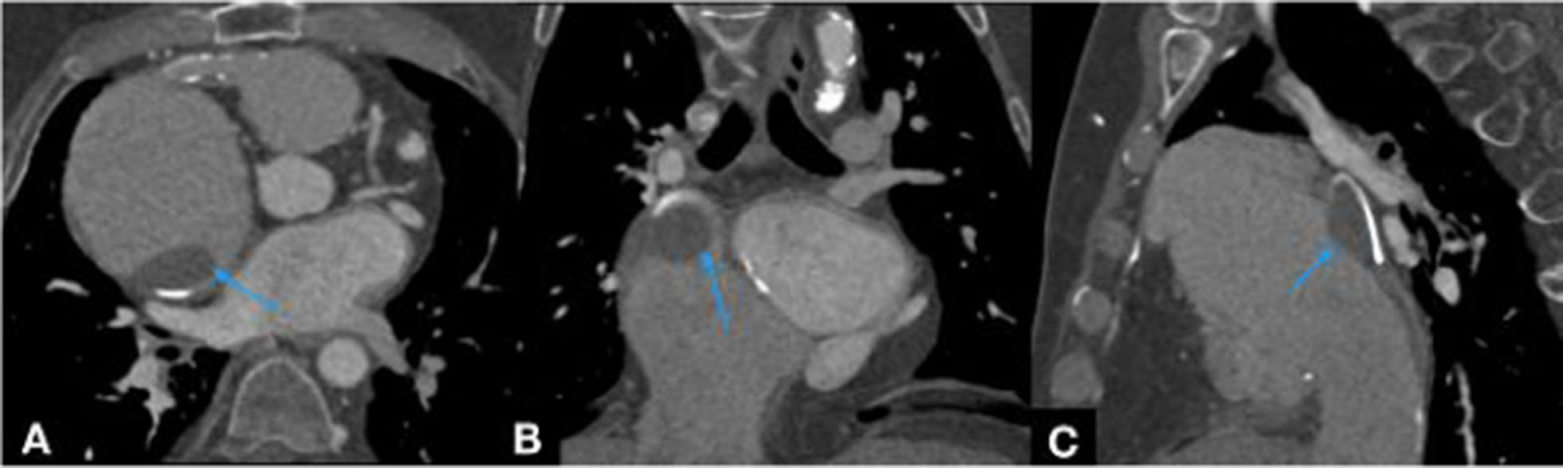
ECG-gated CT study of an atriopulmonary Fontan circulation performed for congenital tricuspid atresia. Axial, coronal and sagittal views in the soft tissue window (**A**–**C**): Large thrombus seen in the right atrium (blue arrows) adhered to the atrial wall. Re-used with permission from Fontan circulation in an adult: A guide for the radiologist. Arzanauskaite M, Nyktari E, Voges I. ESTI-ESCR 2018 / P-0103

**Fig. 24:**
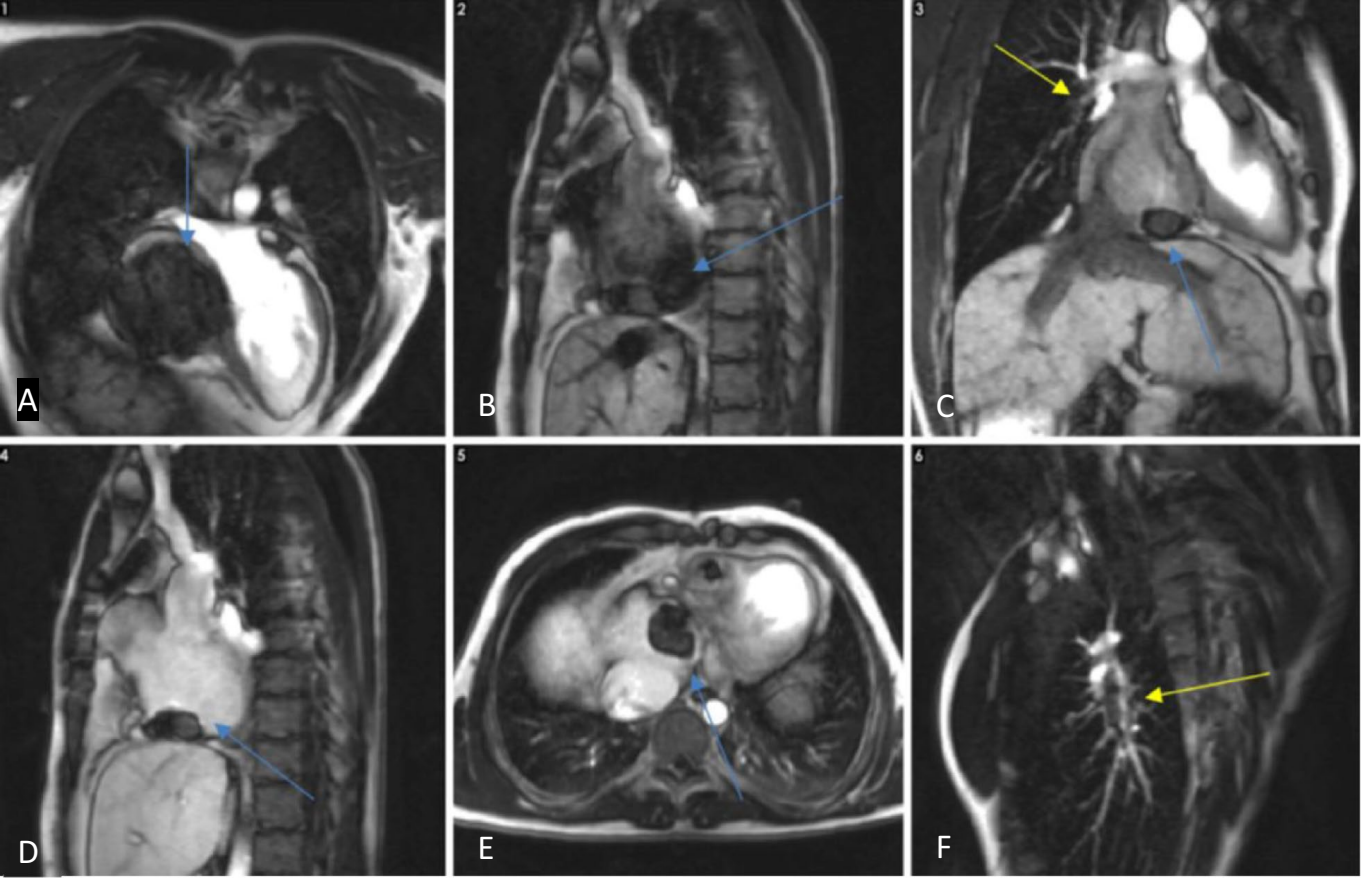
Horizontal long-axis (**A**), sagittal (**B**, **D**), coronal (**C**), axial (**E**) and right pulmonary artery cross-cut views (**F**) of a cardiovascular MRI study in an atriopulmonary Fontan circulation performed for congenital tricuspid atresia. There are filling defects in keeping with thrombi in the right atrium (blue arrows) and in the right pulmonary artery (yellow arrows) on inversion recovery early-phase gadolinium sequences. Note that thrombus appears as very hypointense. Re-used with permission from Fontan circulation in an adult: A guide for the radiologist. Arzanauskaite M, Nyktari E, Voges I. ESTI-ESCR 2018/P-0103

For thrombus detection for all three types of repaired complex ACHD, CT angiography will show filling defects within the cardiac chambers and pulmonary vasculature, which do not enhance (Fig. 23) [60]. MRI can readily identify thrombi within the first minute following the administration of contrast. Delayed enhancement images using SSFP sequences can be performed as an inversion recovery sequence with the TI set to 500–600 ms. This results in thrombus appearing very hypointense compared to surrounding tissues which contain gadolinium (Fig. 24). Advanced sequences that do not use contrast or MRI pulmonary angiography can also allow localisation of the thrombus [31, 35].

## Conclusion

Basic knowledge and understanding of the most common appearances of the heart after repair in complex ACHD is a crucial first step in analysing images presented to the radiologist unfamiliar with repaired complex ACHD. Furthermore, knowledge of basic protocols used in CT and MRI allows for optimal and targeted assessment of relevant complications. By implementing a systematic checklist using key anatomical areas to identify the most common complications in repaired complex ACHD, the most relevant findings can then be reported in a meaningful manner that helps answer the referring clinician’s question. Table 3 summarises the complications discussed, organised by anatomical review areas. By the end of this article, the reader should have a basic understanding of repaired complex ACHD and feel encouraged to refer to the references in text to further advance their knowledge in this challenging area of radiology.

**Table 3:** Summary of complications by anatomical review area and repaired complex ACHD type

Review areas	ACHD type	Pathophysiology	Key imaging features (CT/MRI)
*Heart*			
Heart failure	Repaired TOF	Right heart failure due to pulmonary valve insufficiency/RVOT obstruction	RV and IVC dilatation Septal flattening
	Repaired D-TGA	Right heart failure and tricuspid regurgitation due to opposing the systemic circulation (atrial switch)	SSFP cine MRI sequences assess structure and function
	Fontan circuit	Right heart failure due to high resistance in the Fontan circuit	MRI late gadolinium enhancement in fibrosis/infarction
*Coronary arteries*			
Coronary stenosis	Repaired D-TGA	Ostial stenosis after coronary translocation (arterial switch)	Ostial stenosis on CT coronary angiogram 3D SSFP/contrast angiography on MRI demonstrates ostial stenosis
*Valves*			
Infective endocarditis	All conditions	Endothelial and valvular disruption due to altered flow haemodynamics	Filling defects, septic emboli Vegetations can be low-to-intermediate signal on MRI
*Aorta*			
Aortic dilatation	All conditions	Multifactorial. Progressive medial degeneration in complex ACHD	Dilated ascending aorta (normal values vary per patient)
*Pulmonary vessels*			
Pulmonary hypertension	All conditions	Volume overload due to a residual shunt. High resistance in Fontan circuit	Dilated pulmonary arteries, peripheral pruning
Collaterals, AVMs	Fontan circuit	Increased pathway resistance	Contrast CT/MRI or 3D SSFP MRI to detect dilated tortuous vessels
*Airway and lungs*			
Airway obstruction	All conditions	Extrinsic compression caused by dilated vessels, and/or cardiomegaly	Direct signs of airway stenosis on CT/MRI. Hyperinflated lungs
Plastic bronchitis	Fontan circuit	Abnormal lymphatic flow results in bronchial cast formation	Bronchial intraluminal opacities, groundglass lung changes on CT
*Post-surgical complications and neo-pathways*	All conditions	Thrombosis. Multiple factors. Atrial arrhythmia is a known cause	Filling defects. Hypointense on MRI during early enhancement
	Repaired TOF	Residual RVOT obstruction, conduit stenosis, pulmonary artery stenosis	Contrast-enhanced CT for direct visualisation of stenosis
	Repaired D-TGA	Baffle stenosis/leaks (atrial switch) Supravalvular/branch pulmonary artery stenosis (arterial switch) Conduit stenosis (Rastelli)	MRI: SSFP cine, 3D SSFP, and contrast angiography can all detect areas of stenosis or leak
	Fontan circuit	Intra-atrial baffle or extra-cardiac conduit stenosis	PC MRI to quantify flow or magnitude of leak

## Data Availability

Not applicable.

## Abbreviations

ACHD: Adult congenital heart disease
BT: Blalock–Taussig
CT: Computed tomography
ECG: Electrocardiogram
GRE: Gradient-recalled echo
HU: Hounsfield unit
IE: Infective endocarditis
IV: Intravenous
IVC: Inferior vena cava
LV: Left ventricle
MIP: Maximum intensity projection
MRI: Magnetic resonance imaging
PAH: Pulmonary arterial hypertension
PC: Phase contrast
PDA: Patent ductus arteriosus
REACT: Relaxation-enhanced angiography without contrast and triggering
ROI: Region of interest
RV: Right ventricle
RVOT: Right ventricular outflow tract
SPC: Systemic-to-pulmonary collaterals
SSFP: Steady-state free precession
SVC: Superior vena cava
TGA: Transposition of the great arteries
TOF: Tetralogy of Fallot
US: United States
VSD: Ventricular septal defect
